# Biocompatible Glycoconjugation Enables Sensitive In Vivo Cell Tracking by PET/CT

**DOI:** 10.1021/acs.jmedchem.6c00538

**Published:** 2026-06-24

**Authors:** Nathan Clemons, Anna S. Thickens, Liudmila Lambert Lepesevich, Zachary T. Rosenkrans, Victor Santoro-Fernandes, Anatoly N. Pinchuk, Eduardo Aluicio-Sarduy, Jason C. Mixdorf, Saritha S. D’Souza, Johnathan Caldon, John Kink, Matthew H. Forsberg, Peiman Hematti, Jonathan W. Engle, Igor Slukvin, Christian M. Capitini, Reinier Hernandez

**Affiliations:** † Departments of Medical Physics and Radiology, 5232 University of Wisconsin School of Medicine and Public Health, 1111 Highland Ave, Madison, Wisconsin 53705, United States; ‡ Wisconsin National Primate Research Center, 5228 University of Wisconsin-Madison, 1220 Capitol Court, Madison, Wisconsin 53792, United States; § Department of Pathology and Laboratory Medicine, University of Wisconsin School of Medicine and Public Health, 1685 Highland Ave, Madison, Wisconsin 53705, United States; ∥ Department of Cell and Regenerative Biology, University of Wisconsin School of Medicine and Public Health, 1111 Highland Ave, Madison, Wisconsin 53705, United States; ⊥ Carbone Cancer Center, University of Wisconsin School of Medicine and Public Health, 600 Highland Ave, Madison, Wisconsin 53792, United States; # Division of Hematology/Oncology, Medical College of Wisconsin, 9200 W. Wisconsin Ave, Milwaukee, Wisconsin 53226, United States; ∇ Department of Pediatrics, University of Wisconsin School of Medicine and Public Health, 600 Highland Ave, Madison, Wisconsin 53792, United States

## Abstract

Cell-based therapies have transformed the treatment landscape for cancer, yet their clinical translation remains limited by unpredictable in vivo behavior and variable patient responses. Accurate, noninvasive image-based tracking of therapeutic cells, such as PET/CT, is essential for understanding biodistribution, improving safety, and optimizing the design of next-generation treatments. However, existing radiolabeling strategies for cell tracking using PET/CT lack the stability and sensitivity required for reliable long-term imaging. Here, we present a direct radiolabeling strategy that oxidizes cell surface sialic acids to conjugate aminooxy-DFO (AOD) and subsequently radiolabels cells with ^89^Zr under biocompatible conditions. We radiolabeled five human and nonhuman primate immune cell types with high radiochemical incorporation (17–194 μCi per million cells) and purity (∼90%), while preserving cell viability. Serial PET/CT imaging over 7–8 days revealed conserved biodistribution patterns across all cell types tested. This approach provides a robust, applicable platform for longitudinal PET/CT tracking of therapeutic cells.

## Graphical abstract

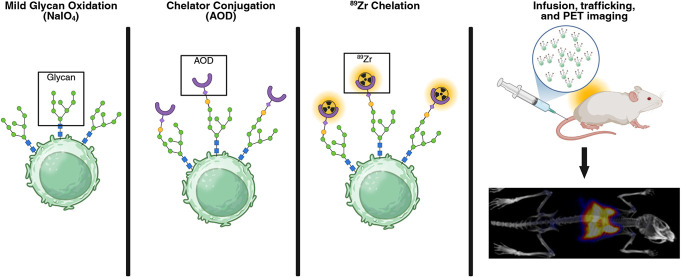

## Introduction

Adoptive cell therapies are Food and Drug Administration (FDA)-approved treatments for cancer and other diseases, including chimeric antigen receptor (CAR) T cells for blood cancers, T cell receptor (TCR) T cells for synovial sarcoma, tumor infiltrating lymphocytes (TILs) for melanoma and mesenchymal stromal cells (MSCs) for gut graft-versus-host-disease (GVHD). Emerging adoptive cell therapies are being developed for other solid tumors, with monocytes or macrophages being explored for inflammatory and degenerative conditions such as rheumatoid arthritis, myocardial infarction, liver sclerosis, and cancer.^1^ T cell-based therapies have shown excellent clinical responses and durable remissions in cancer.^2–6^ In the field of hematopoietic cell transplantation, peripheral blood stem cells, bone marrow and umbilical cord blood contain hematopoietic progenitor cells (HPCs), monocytes, macrophages, lymphocytes, and granulocytes are useful for treating nonmalignant hematopoietic disorders and immunodeficiencies.^7–11^ Other cell products including autologous fibroblasts and chondrocytes are being developed for soft tissue and cartilage repair.^12,13^

Despite the clinical success of adoptive cell therapies, widespread adoption remains limited due to an incomplete understanding of therapeutic mechanisms, off-target toxicities, and insufficient in vivo trafficking and persistence, with many therapies stalled in early phase studies due to highly inefficient safety and pharmacological evaluation methodologies.^14^ These parameters are critical for guiding therapeutic optimization, FDA approval, and ultimately clinical translation. Molecular imaging, particularly positron emission tomography/computed tomography (PET/CT), can address this need by enabling temporally resolved, quantitative whole-body tracking of therapeutic cells in vivo with a detection sensitivity unparalleled by other anatomical modalities, such as MRI.^15–17^

Cell radiolabeling with positron-emitting radionuclides (e.g., ^18^F, ^68^Ga, ^64^Cu, or ^89^Zr) is a prerequisite for PET/CT tracking, but current approaches have significant limitations. Direct methods include metabolic uptake (^18^F-FDG), passive diffusion loading (^89^Zr-oxine), chelator conjugation to cell surface proteins (e.g., DFO-NCS), or phagocytosis of radiolabeled nanoparticles.^18–21^ The most common, ^89^Zr-oxine and DFO-NCS, suffer from toxicity, poor radiochemical incorporation, and loss of label in vivo, leading to nonspecific and confounding imaging results.^22,23^ In DFO-NCS approaches using prelabeling with ^89^Zr, the reactive isothiocyanate group can be hydrolyzed, further reducing incorporation. Overall, an improved strategy is needed that enhances radionuclide incorporation and stability while maintaining cell viability and function.

To address this need, we targeted cell surface glycans, which are abundantly and ubiquitously expressed on all mammalian cell types, though their specific composition and expression levels can vary.^24^ Glycans, including glycosphingolipids, proteoglycans, and O- or N-linked glycoproteins, play roles in structure, communication, immunity, and cancer, and their high abundance makes them ideal anchoring points for covalent conjugation.^25,26^ Additionally, glycoconjugations are generally stable, safe, and nondisruptive to cell function.^27,28^

In this report, we introduce a three-step direct radiolabeling technique that covalently conjugates a derivative of deferoxamine (DFO), a well-studied ^89^Zr chelator, to sialic acids on the cell surface.^26,29^ The process involves selective oxidation of sialic acids, conjugation of aminooxy-DFO (AOD), and subsequent radiolabeling with ^89^Zr under biocompatible conditions. To evaluate this approach’s robustness and potential for clinical impact, we tested its radiochemical incorporation, maintenance of cell viability and function, and stability. We further evaluated the potential of quantitative PET/CT tracking of multiple human and nonhuman primate (NHP) cell types, including monocytes, peripheral blood-derived mononuclear cells (PBMCs), neutrophils, and T cells, using this three-step direct cell radiolabeling approach.

## Results

### Aldehyde-Reactive Aminooxy-DFO

To enable conjugation with aldehydes generated from sialic acid oxidation, the amino group in the chelator DFO was functionalized with (Boc-aminooxy)­acetic acid to introduce an aminooxy reactive group (Figure 1A) and subsequently conjugated to oxidized sialic acids (Figure 1B). Compound **1** was isolated by silica gel chromatography as a yellow solid in high purity (>99%) and yield (98% yield, 226.7 mg). AOD was then obtained following Boc deprotection with trifluoroacetic acid (TFA) at 52% yield and high purity. ^1^H NMR, ^13^C NMR and mass spectrometry for both **1** and AOD conformed with the structures (Figures S1–S6). AOD was further characterized by analytical HPLC (Figures S7, S8) prior to in vivo studies.

**Figure 1:**
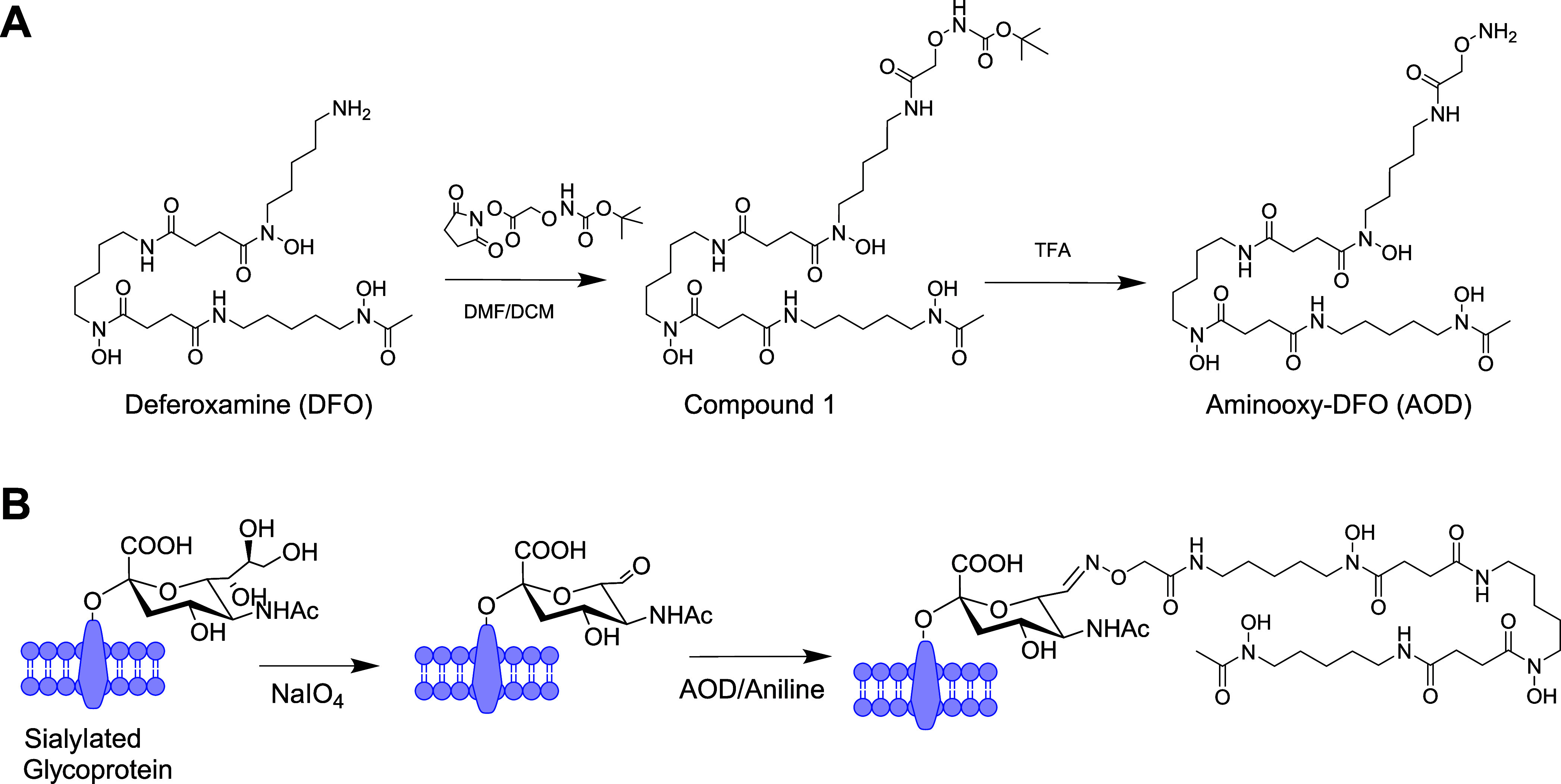
Schematic representation of (A) synthesis of aldehyde-reactive deferoxamine bifunctional chelator and (B) subsequent conjugation to oxidized sialic acids.

### Optimization of Glycoconjugation

Conditions for the periodate oxidation of sialic acids on the cell surface and subsequent ligation with AOD were optimized using U937 cells (Figure 2). One mM NaIO_4_ in pH 7.4 PBS at room temperature for 20 min resulted in no significant loss of viability and was therefore used for subsequent radiolabeling experiments (Figure 2A). 50–500 μM AOD with 10 mM aniline for 30 ± 10 min was determined to be the least detrimental to cell viability and also used subsequently (Figure 2B). Notably, all conjugation reactions were carried out in pH 6.5 PBS to promote the nucleophilic character of AOD. Regarding the dependence of the catalyst aniline in the conjugation step, upon radiolabeling with a limited amount of ^89^Zr (<400 μCi for 3–4 × 10^6^ cells), both the radioactive incorporation (μCi per million cells) and cell viability after the radiolabeling were shown to be significantly lower in the – aniline group (*p* = 0.0280 and 0.0428, respectively), whereas radiochemical purity was not affected (*p* = 0.9542, Figure 2C, 2D, and 2E).

**Figure 2:**
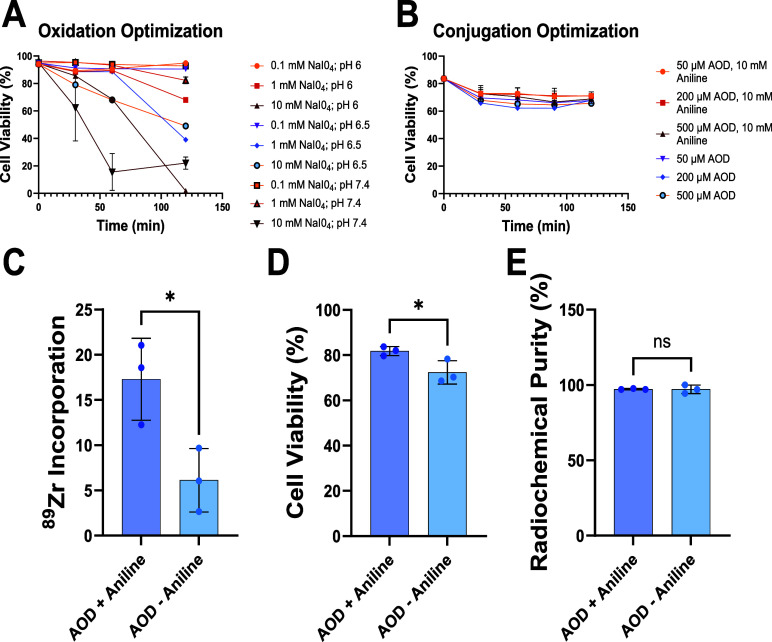
Optimization of the oxidation and conjugation steps. A) Optimization of the time, pH, temperature, and [NaIO_4_] of the sialic acid oxidation (*N* = 3 per group, starting viability: 94%). B) Optimization of the time, [AOD], and presence of aniline of the conjugation reaction (*N* = 3 for AOD + aniline groups, *N* = 1 for AOD-only groups, starting viability: 84%). C) ^89^Zr incorporation (μCi per million cells) in U937 cells conjugated in the presence or absence of aniline (*N* = 3 per group). D) Post-radiolabeling viability of [^89^Zr]­Zr–U937 cells conjugated in the presence or absence of aniline (*N* = 3 per group). E) Radiochemical purity of [^89^Zr]­Zr–U937 cells conjugated in the presence or absence of aniline (*N* = 3 per group). * denotes *p* < 0.05, ns denotes *p* > 0.05.

Therefore, the presence of aniline (10 mM) was determined to be essential for the efficient conjugation of AOD to reactive sialic acids generated in oxidation step, in strong accordance with previous studies.^26^ Final optimized conjugation protocols included cell incubation with NaIO_4_ (1 mM) for 20 min in PBS (pH 7.4), washing and resuspension of oxidized cells into 1 mL PBS (pH 6.5), incubation with AOD (200 μM) in aniline (10 mM) for 40 min, and subsequently washing to remove unconjugated AOD. Centrifugation (200–600*g*, 5 min) was used for each washing step. These conditions were then applied across a range of immune cell types, with labeling performance summarized (Table 1).

**Table 1:** Radiolabeling Characterization^a^ ^b^

Group	Cell Type	Purity (%)	Incorporation (μCi/10^6^ cells)	Viability preradiolabeling (%)	Viability post-radiolabeling (%)
Human	U937 Cells	92 ± 1	194 ± 64	78 ± 9	77 ± 12
Human	PBMCs	99	107	44	61
NHP	Neutrophils	99	64	94	92
NHP	T cells	95	17	93	93
Human	Jurkat T cells	84	133	88	76

a Each radiolabeled cell type was characterized by average purity radiochemical incorporation, and cell viability before and after the radiolabeling.

b PBMC = peripheral blood mononuclear cell; NHP = nonhuman primate.

### Radiolabeling, Stability and Functional Assessment of [^89^Zr]­Zr–U937 Cells

Using the optimal conjugation conditions, radiolabeling purity was assessed in vitro using the U937 human monocytic leukemia cell line. Sample iTLC lanes were used to acquire the average purity (Figure 3A and 3B). The reaction mix is a sample acquired after the radiolabeling reaction, showing all species in solution. However, upon multiple centrifugation steps, [^89^Zr]­Zr-AOD and free ^89^Zr are washed out of solution, leaving a ≥ 92% pure solution of [^89^Zr]­Zr–U937 cells in all radiolabeling experiments. In the stability assay, more than 95% purity and 80% viability were observed in all samples across time (*N* = 4, Figure 3C and 3D). Finally, unlabeled U937 cells and [^89^Zr]­Zr–U937 cells were assayed for phagocytic activity using a flow cytometry assay with latex beads coated with GFP-labeled rabbit IgG (Figure 3E, 3F, and 3G). Both unlabeled U937 cells (Figure 3E) and [^89^Zr]­Zr–U937 cells (Figure 3F) showed more than 90% GFP-associated fluorescence compared to autofluorescence controls (Figure 3E), indicating that the radiolabeling procedure does not inhibit phagocytic activity in vitro.

**Figure 3:**
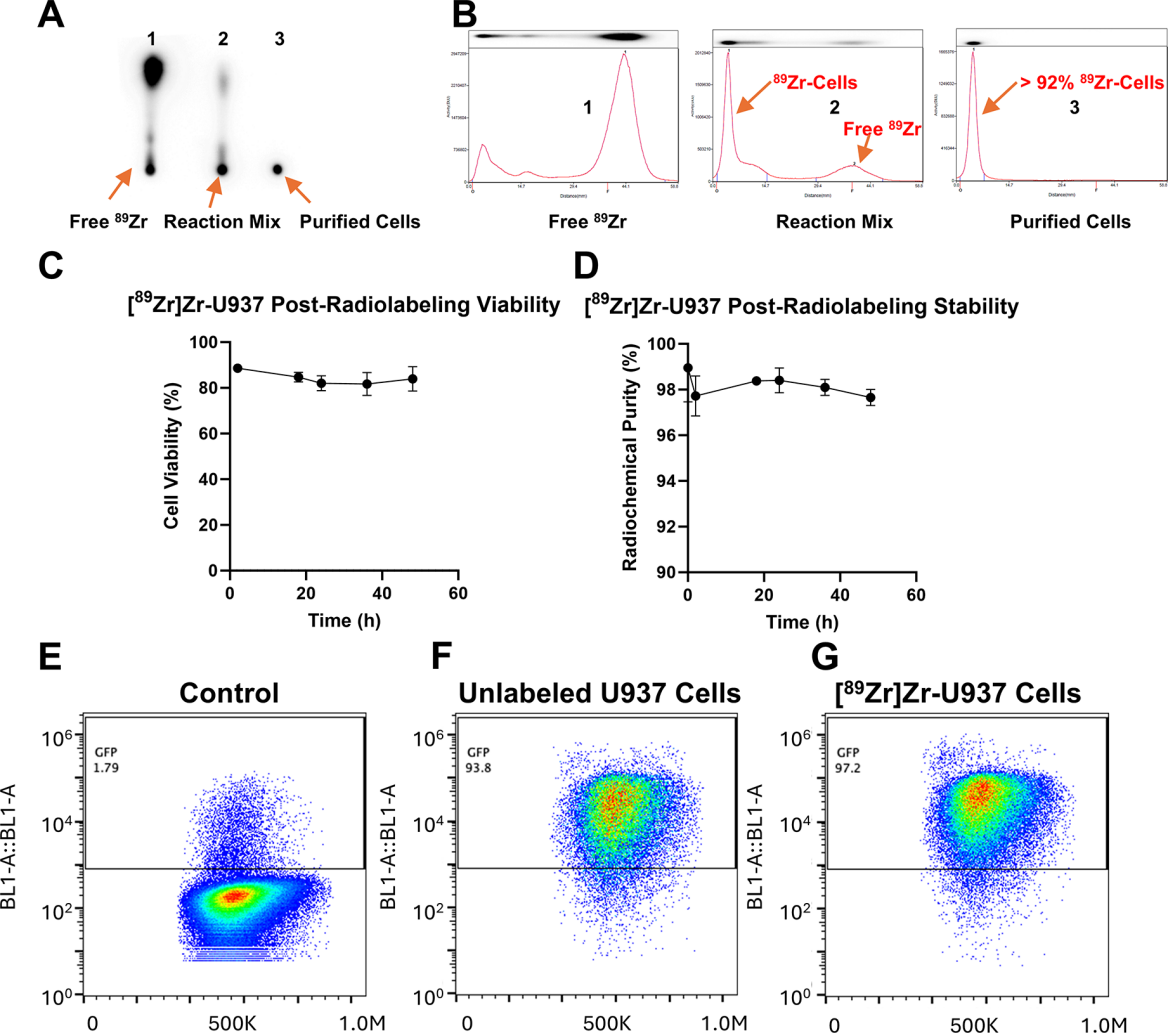
[^89^Zr]­Zr–U937 cells radiolabeling. A) iTLC lanes depicting free ^89^Zr (1), the reaction mixture (2), and purified [^89^Zr]­Zr–U937 cells (3). B) iTLC profiles of free ^89^Zr (1), the reaction mixture (2), and purified [^89^Zr]­Zr–U937 cells (3). C) [^89^Zr]­Zr–U937 cells post-radiolabeling in vitro cell viability over 48h (*N* = 4). D) [^89^Zr]­Zr–U937 cells post-radiolabeling radiochemical purity over 48h (*N* = 4). Assessment of phagocytosis by flow cytometry for E) Unlabeled U937 cells separated by autofluorescence (1.79% of total population), F) unlabeled U937 cells incubated with fluorescent beads for 30 min separated by GFP-associated fluorescence (93.8%), and G) [^89^Zr]­Zr–U937 cells incubated with fluorescent beads for 30 min separated by GFP-associated fluorescence (97.2%).

Radiolabeling of U937 cells with ^89^Zr demonstrated unusually high per-cell radioactivity incorporation (up to 194 μCi per million cells), with high purity (92%) and cell viability (93%). Overall, the radiolabeling procedure showed robust radiochemical yields across cell types (Table 1), while maintaining cell viability after the completion of the protocol.

### In Vivo Cell Tracking Using PET/CT

[^89^Zr]­Zr–U937 cells were adoptively transferred to NOD-SCID-γc^–/–^ (NSG) mice (*N* = 3, 126–150 μCi, 3.5–4.1 × 10^5^ cells) and imaged by serial PET/CT from 3 h to 8 days post-injection. Maximum intensity projection (MIP) PET/CT images were obtained of [^89^Zr]­Zr–U937 cells (Figure 4A). ROI analyses of the images (Figure 5A) confirmed elevated cellular trafficking in the lungs (19.9 %IA/g) and liver (23.7 %IA/g) at 3 h (*N* = 2; data for mouse 3 were lost due to data corruption), with a subsequent decrease in lung uptake to 2.4 ± 0.5 %IA/g and redistribution to the liver (31.5 ± 0.8 %IA/g) and spleen (13.2 ± 0.6 %IA/g) at 24 h post-injection. Liver and spleen uptake remained stable through day 8 (30.0 ± 2.2 %IA/g and 12.2 ± 0.6 %IA/g, respectively). Bone uptake at 8 days (1.4 ± 0.3 %IA/g) was low when compared to literature values of free [^89^Zr]­Zr-oxalate (∼17% at 6 d) and free ^89^Zr leached from [^89^Zr]­Zr-Oxine (4–8% at 2 d–7 d).^30–32^ The low bone uptake and a stable whole-body signal indicate excellent in vivo stability of the radiolabel with minimal excretion or release of free ^89^Zr.

**Figure 4:**
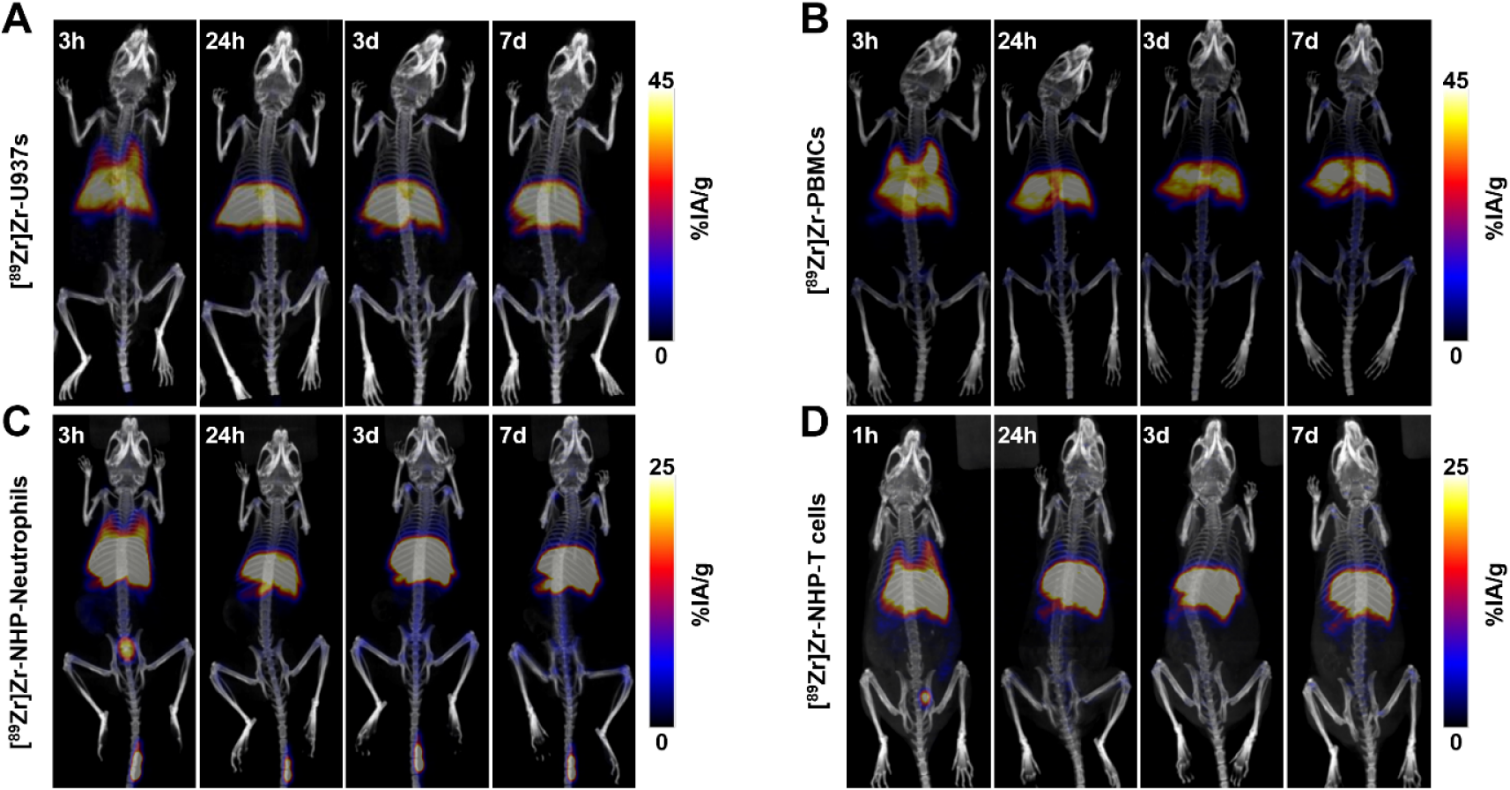
Representative maximum intensity projections (MIPs) of PET/CT images of radiolabeled cells. Four cell types were administered into NSG mice: A) [^89^Zr]­Zr–U937 cells (*N* = 3), B) [^89^Zr]­Zr-PBMCs (*N* = 2), C) [^89^Zr]­Zr-NHP-Neutrophils (*N* = 3), and D) [^89^Zr]­Zr-NHP-T cells (*N* = 2). All cell populations showed similar distribution to the lung and liver at early time point and the liver and spleen at day 7 post administration.

**Figure 5:**
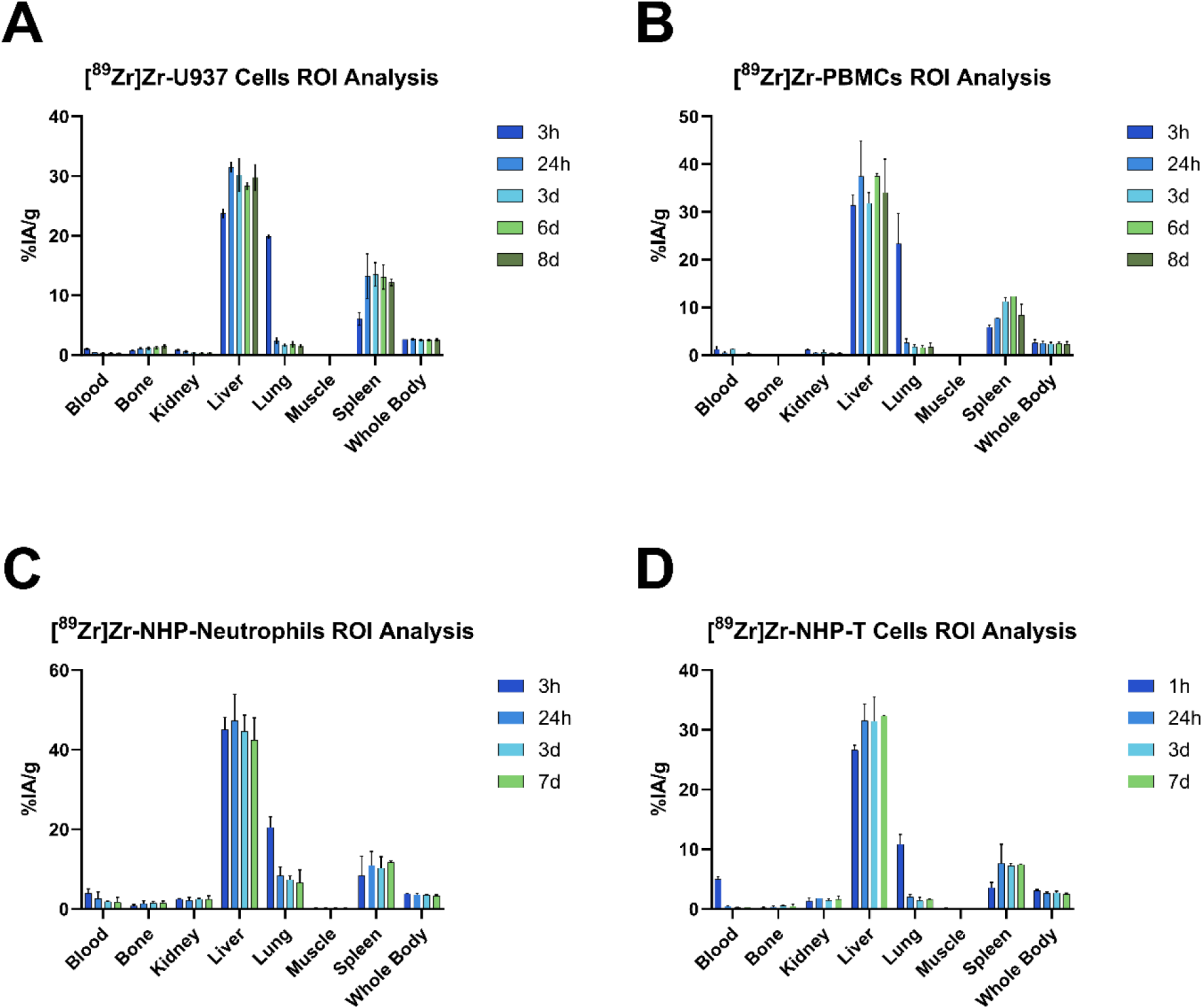
Region of Interest (ROI) analysis of PET/CT imaging. A) [^89^Zr]­Zr–U937 cells. B) [^89^Zr]­Zr-PBMCs. C) [^89^Zr]­Zr-NHP-Neutrophils. D) [^89^Zr]­Zr-NHP-T cells.

Human PBMCs were then radiolabeled with ^89^Zr (90 μCi per million cells) and had a similarly high purity (93%), with preserved viability. PET/CT MIPs of NSG mice receiving 16–21 μCi [^89^Zr]­Zr-PBMCs (1.3–1.7 × 10^5^ cells) corroborated the anticipated early trafficking to the lungs (23.4 %IA/g) and liver (31.4 %IA/g), despite the heterogeneous cell composition (Figure 4B). By day 8, liver uptake persisted (34.1 %IA/g), spleen uptake increased (8.4 %IA/g), and lung uptake decreased (1.7 %IA/g). Whole-body signal declined slightly from 2.8 %IA/g at 1 h to 2.3 %IA/g at 8 days, indicating minimal activity excretion (Figure 5B).

To determine if there were species specific effects, nonhuman primate (NHP) neutrophils and T cells were also evaluated. Induced pluripotent stem cell (iPSC)-derived NHP neutrophils were radiolabeled (64 μCi per million cells) with high purity (99%) and viability (92%). In comparison, PBMC-derived NHP T cells were labeled with moderate efficiency (17 μCi per million cells) but with high purity (95%) and viability (93%). NSG mice injected with [^89^Zr]­Zr-NHP-Neutrophils (85 ± 8 μCi, 1.3 ± 0.1 × 10^6^ cells) displayed initial lung (20.5 ± 2.6 %IA/g) and liver (45.0 ± 3.2 %IA/g) trafficking at 3 h, followed by redistribution to the liver (42.4 ± 5.7 %IA/g) and spleen (11.7 ± 0.5 %IA/g) by day 7 (Figure 4C, Figure 5C). Two mice received [^89^Zr]­Zr-NHP-T cells (14 μCi, 8 × 10^5^ cells) and showed early lung (10.9 %IA/g) and liver (26.7 %IA/g) trafficking, later redistributing to the liver (32.4 ± 0.1 %IA/g) and spleen (7.5 ± 0.1 %IA/g) by day 7 (Figure 4D, Figure 5D).

Finally, Jurkat T cell leukemia cells were radiolabeled (133 μCi per million cells) and injected into an NSG mouse (69 μCi, 5 × 10^5^ cells). [^89^Zr]­Zr-Jurkats trafficked to the lungs (17.9 %IA/g) and liver (38.2 %IA/g) at 1 h, redistributing to the liver (35.2 %IA/g) and spleen (5.8 %IA/g) by day 7 (Figure S9, Figure S10). Unlike other cell types, Jurkat cells exhibited persistent bone uptake (1.6 %IA/g at both 1 h and 7 days; Figure S10), perhaps indicating tissue tropism of T cells versus other cell types.

[^89^Zr]­Zr-DFO exhibited a markedly different biodistribution from the radiolabeled cells. It rapidly cleared renally within 24 h, accumulating primarily in the bladder, with no detectable lung, liver, or spleen uptake; ex vivo biodistribution was not performed due to complete clearance (Figure S16, Figure S17, and Figure S18). PET-derived activity measurements for all cell types were obtained (Figure 5), with detailed ROI quantification provided (Tables S1–S6).

## Discussion and Conclusions

Over the last few decades, tracking living cells after adoptive transfer using nuclear medicine imaging modalities has proven valuable, both preclinically for the development of therapeutic cell products and clinically for the detection of infections using radiolabeled white blood cells.^33,34^ Despite tremendous progress in cell therapies with the advent of CAR T cells, TCR T cells, TILs and other immune cells in oncology, and MSCs in GVHD, the development of an FDA-approved radiolabeling agent for cell-tracking has lagged due to a lack of efficient radiolabeling techniques that enable sensitive detection of small numbers of adoptively transferred cellular populations within target tissues or tumors. Direct ex vivo radiolabeling methods attach isotopes directly to cells for PET or Single Photon Emission Computed Tomography (SPECT), while indirect methods genetically engineer cells to express reporter proteins or receptors.^35^ Direct labeling is simpler, robust, and has a clearer regulatory path, making it ideal for nonproliferative cells such as monocytes and macrophages, where signal dilution from cell division is minimal. Its limitations include a finite imaging window determined by isotope half-life and cell clearance. Indirect labeling, while suited for highly proliferative cells like hematopoietic stem cells,^17,36,37^ is more complex and costly, with greater regulatory hurdles.^35,36^

Because PET and SPECT imaging modalities rely on gamma emission detection, the sensitivity for detecting labeled cells is directly proportional to the amount of activity incorporated into each cell. Current ex vivo radiolabeling methodologies achieve relatively low per-cell activity incorporation on the order of <13.5 μCi per million cells, with many approaches yielding <1 μCi per million cells.^18,20–23,38^ Low radiochemical incorporation therefore yields poor PET signal, limiting PET/CT detecting sensitivity and the ability to resolve small structures within organs of interest, such as the lungs, liver and bone marrow. Herein, we developed a highly efficient biocompatible cell labeling methodology that suggests a substantial improvement in activity incorporation and thus PET/CT sensitivity, though formal head-to-head comparisons will be needed to further elucidate this enhancement.

Our radiolabeling protocol involved the oxidation of vicinal diols in sialic acid to aldehydes (optimal conditions: 1 mM NaIO_4_, pH 7.4 PBS, 20 min) and conjugation of the widely employed ^89^Zr chelator DFO (50–500 μM AOD, 10 mM aniline, 40 min) while preserving cell viability (Figure 2). Similar conjugation methodologies were reported successful for the stable conjugation of fluorescent dyes to cells with excellent biocompatibility.^26^ [^89^Zr]­Zr–U937 cells demonstrated excellent radiochemical purity (>95%) and maintained viability (>80%) for up to 48 h post-radiolabeling (Figure 3). Notably, phagocytic function was preserved, as [^89^Zr]­Zr–U937 cells and unlabeled U937 cells exhibited similar phagocytic capacity when assayed by flow cytometry. We then used the optimized protocol to efficiently radiolabel PBMCs, NHP-Neutrophils, NHP-T cells, and Jurkat T cells while maintaining viability (Table 1), enabling intravenous adoptive transfer and longitudinal PET/CT imaging in mice (Figure 4). All populations, including those with a heterogeneous mixture of immune cells (PBMCs), showed similar biodistribution with initial lung and liver accumulation followed by redistribution to the liver and spleen at day 7 (Figure 5), aligning with previous reports for the tracking of these immune cell types using established methods.^18,20–23,38^ Ex vivo biodistribution corroborated PET/CT ROI data, confirming the accuracy of the imaging quantification (Figures S11–15, Table S7). The reduced viability of the PBMCs was consistent with donor-to-donor heterogeneity and the sensitivity of human PBMCs, while the final [^89^Zr]­Zr-PBMCs remained enriched for viable cells.^39,40^

Our protocol’s highly effective radioactive cell incorporation and stability offer key advantages. Covalent linkage of AOD to surface sialic acids minimizes radioactive losses compared to passive diffusion or protein conjugation paradigms, as [^89^Zr]­Zr-AOD remains covalently bound to abundant membrane sialic acids. The aminooxy group was chosen over alternative aldehyde-reactive functionalities, including hydrazine-based groups, due to rapid oxime ligation kinetics at physiological pH in the presence of the aniline catalyst and the enhanced hydrolytic stability of oxime versus hydrazone bonds at physiological pH.^41^ DFO was used because it is a natural siderophore and well characterized chelator of +3/4 metals.^42,43^ Unlike ^18^F-FDG-, ^89^Zr/^111^In-oxine, or nanoparticle-based methods, our approach offers improved radiochemical stability as covalently anchored ^89^Zr is less likely to be dissociated or effluxed from the cell, even following internalization.^21,44–46^ Additionally, radiolabel stability, cell viability, and phagocytic function were preserved in [^89^Zr]­Zr–U937 cells over 48 h in culture, capturing the period of highest radiation exposure and confirming that high radiochemical incorporation does not compromise cell integrity. Moreover, compared to reported NCS-DFO, which nonspecifically modifies proteins through free amines, our method selectively targets the cell glycocalyx, reducing the risk of significantly altering cell trafficking or function. High incorporation also enables radiolabeling of a small fraction of the cells as an imaging surrogate population (<10%), preserving the efficacy of the overall treatment while still providing crucial biodistribution data. To this end, an aliquot of cells can be separated prior to administration, radiolabeled as described, and then added back into the full cell injection. Although chemical modification could slightly alter trafficking, this small labeled subset remains valuable for longitudinal whole-body tracking, overcoming the practical limitations of blood sampling and flow cytometry for determining cell biodistribution.^47–49^

While incorporation is crucial for PET image quality, retention, cell viability, and biological relevance are equally important. High activity per cell is essential to achieve clear PET signals, especially when tracking small populations in vivo. ^89^Zr-oxine labeling typically yields 0.2–3 μCi per million cells,^20,22,23^ and suffers from low retention and cytotoxicity at higher doses. Chelator-based strategies like [^89^Zr]­Zr-DBN^21^ and [^89^Zr]­Zr-DFO^38^ reach 1.6–13.5 μCi per million cells, while [^18^F]-FDG^18^ or [^111^In]­In-oxine^32^ achieve 5–80 μCi per million cells but suffer from rapid efflux and, for ^18^F, a short half-life. The method introduced here achieves 17–194 μCi per million cells, which compares favorably to published values for existing direct cell radiolabeling approaches. Although not head-to-head comparisons, these data demonstrate that our method provides a balance of high incorporation, retention, and biocompatibility, enabling sensitive and durable tracking of adoptively transferred cells.

Although our method achieves best-in-class benchmarks in terms of radiochemical yields and biocompatibility, several challenges remain before translation to clinical testing. Similar to other direct labeling methods, PET imaging alone cannot distinguish between signal originating from viable labeled cells and signal arising from secondary uptake by host phagocytes following cell death. Thus, PET signal may not be fully representative of the live cell population. However, relatively stable whole body PET signal observed across all cells tested may indicate that ^89^Zr was not leached from the radiolabeled cells. To confirm in vivo stability, however, tissue-level validation, such as autoradiography combined with immunostaining for transferred cell-specific markers, would help determine whether retained radioactivity remains associated with the transferred cells. In follow-up studies, we will coinject unlabeled cells and perform ex vivo analyses including autoradiography, flow cytometry, and bioluminescence tagging to cross-validate our imaging findings.^50,51^ Additionally, the small sample size employed in these cohorts limits the quantitative interpretation of the imaging data; these results provide proof-of-concept radiolabeling feasibility and versatility across diverse cell types rather than characterizing biodistribution. Larger cohorts will be required for quantitative biodistribution analysis and are planned for future studies focused in the particular cell type of interest. Chemical modification of sialic acids may also alter trafficking, which is a limitation shared with other chelator-based methods like NCS-DFO. Finally, variability in labeling among cell types likely reflects differences in surface sialic acid expression,^29^ which can change during differentiation or with culture conditions.^52,53^

In conclusion, our approach achieves best-in-class radiochemical incorporation with preserved viability and function across diverse human and NHP cell types, enabling sensitive in vivo cell tracking using PET/CT in murine models. The ubiquity of sialic acids across mammalian cell membranes makes this strategy broadly applicable to any therapeutic cell type. Future studies will compare this approach to existing methods and extend the approach to therapeutic cells such as CAR-T cells and to large animal models, generating pharmacology/toxicology data to support translational efforts to validate this approach in a human clinical trial.

## Experimental Section

### Reagents

Deferoxamine mesylate salt was purchased from Macrocyclics Inc. (Dallas, TX). Unless otherwise specified, all reagents and solvents were obtained from Thermo Fisher Scientific (Waltham, MA) or Sigma-Aldrich (Milwaukee, WI) and used without further purification. TraceSELECT water (Honeywell, Muskegon, MI) was used for all aqueous solutions unless otherwise noted. All compounds were ≥95% pure by UV/vis HPLC, except AOD, which was determined to be 92% pure.

^89^Zr was produced on a GE PETtrace biomedical cyclotron by irradiating natural yttrium foils with 16.2 MeV protons, followed by purification via solid-phase chromatography and elution with 1 M oxalic acid.

### Cell Culture

U937 human pro-monocytic cells and the Jurkat T cells line were purchased from ATCC (Manassas, VA) and cultured in RPMI media supplemented with l-glutamine and 10% fetal bovine serum (Life Technologies Corporation, Carlsbad, CA). ATCC guidelines were followed for cell authentication using morphology monitoring, growth curve analysis, and testing for mycoplasma within 6 months of use. Cells were maintained at 37 °C and 5% CO_2_, passaged every 24–48 h, and kept below passage 30 for all in vivo experiments.

Human primary nonmobilized PBMCs were obtained from healthy donor peripheral blood using an institutional review board (IRB)-approved protocol (2017–0170) and isolated using the MACSprep PBMC Isolation Kit (Miltenyi Biotec, San Jose, CA) without density gradient centrifugation.

Hematopoietic differentiation from NHP iPSCs was performed as previously described.^54,55^ For neutrophil differentiation, hematopoietic progenitors were cultured in α-MEM medium with 10% FBS and 50 μM β-ME supplemented with 150 ng/mL of G-CSF, 20 ng/mL of SCF, 20 ng/mL of IL-6 and 10 ng/mL of IL-3 for 7 days. The neutrophil phenotype was confirmed by flow cytometry and cytospin analysis.

For T cell isolation, peripheral blood was diluted 1:1 with PBS-EDTA and subjected to density gradient centrifugation to isolate mononuclear cells (MNCs). The MNCs were washed twice with PBS-EDTA and labeled with CD14, CD19, CD11b and CD56 antibodies, all tagged to APC fluorochrome. The cells were washed, stained with APC microbeads and CD3 positive cells were negatively selected by magnetic activated cell sorting. The enrichment of CD3 positive cells was confirmed by flow cytometry. The sorted cells were plated for 24 h in RPMI medium with 5% FBS and used for labeling the next day.

### Animals

Eight to 16-week-old male and female NOD-SCID-γc^–/–^ (NSG) mice (Jackson Laboratories, Bar Harbor, ME) were used in this study. NSG mice were bred in an animal vivarium at the University of Wisconsin-Madison. All mice were housed and cared for following the Guide for the Care and Use of Laboratory Animals. All animal experiments were approved by the University of Wisconsin-Madison Institutional Animal Care and Use Committee (IACUC) under protocol M005915.

### Synthesis of Aldehyde-Reactive Aminooxy-DFO

#### Compound **1**

N-Boc-aminooxy acetic acid N-hydroxysuccinimide ester (100.2 mg, 0.347 mmol) and deferoxamine mesylate salt (207.5 mg, 0.316 mmol) were dissolved in dimethylformamide (8 mL) and triethylamine (86 μL, 0.62 mmol) was added. The reaction mixture was stirred overnight at 60 °C and analyzed by TLC for completion (TLC solvent system CHCl_3_–MeOH–NH_4_OH (10:8:1)). The solvent was evaporated at 50 °C (water bath temperature). The crude product was purified by silica gel chromatography (CHCl_3_–MeOH, gradient: 95:5 to 80:20 v/v) to obtain 226.7 mg (98% yield) of a light-yellow solid.

^1^H NMR (400 MHz, DMSO-d_6_): δ 10.29 (s, 1H), 9.78–9.46 (m, 3H), 8.31 (s, 1H), 7.98 (s, 1H), 7.77 (t, *J* = 5.7 Hz, 2H), 4.13 (s, 2H), 3.45 (t, *J* = 7.1 Hz, 6H), 3.10 (q, *J* = 6.7 Hz, 2H), 3.00 (q, *J* = 6.6 Hz, 4H), 2.57 (t, *J* = 7.4 Hz, 4H), 2.26 (t, *J* = 7.3 Hz, 4H), 1.96 (s, 3H), 1.56–1.45 (m, 2H), 1.44–1.32 (m, 2H), 1.41 (s, 9H), 1.26–1.18 (m, 6H).

^13^C NMR (101 MHz, DMSO) δ 178.64, 174.61, 168.26, 162.81, 81.16, 75.24, 47.53, 40.59, 40.38, 40.17, 39.96, 39.75, 39.54, 39.33, 38.88, 36.26, 31.25, 29.27, 29.18, 28.44, 28.39, 26.49, 23.95, 20.81.

**MS (ESI** ^**+**^ **):** *m*/*z* calculated for C_32_H_60_N_7_O_12_ [M + H]^+^: 734.430; found: 734.429; calculation for C_32_H_59_N_7_NaO_12_ [M + Na]^+^: 756.412; found: 756.409.

#### Aminooxy-DFO (AOD)

Compound **1** (226.7 mg, 0.309 mmol) was dissolved in CHCl_3_ (5 mL) and trifluoroacetic acid (2 mL) was added. The reaction mixture was stirred overnight at room temperature. The reaction was analyzed by TLC (CHCl_3_–MeOH–NH_4_OH (90:10:1), Rf = 0.45 for the target compound). The crude product was purified by silica flash chromatography (CHCl_3_–MeOH, gradient: 95:5 to 80:20 v/v) to give Aminooxy-DFO (AOD) as a white powder (52% yield, 101.8 mg). AOD was confirmed to be 92% pure by HPLC analysis (Figure S8). However, the low UV absorptivity of AOD underestimates the purity observed by HPLC.

^1^H NMR (400 MHz, DMSO-d_6_): δ 9.62 (d, *J* = 18.0 Hz, 3H), 7.81–7.69 (m, 3H), 4.30 (s, 0H), 3.93 (s, 1H), 3.45 (t, *J* = 7.0 Hz, 6H), 3.08 (q, *J* = 6.3 Hz, 2H), 3.00 (q, *J* = 6.6 Hz, 4H), 2.57 (t, *J* = 7.3 Hz, 4H), 2.26 (t, *J* = 7.4 Hz, 4H), 1.86 (s, 1H), 1.80 (s, 1H), 1.54–1.45 (m, 6H), 1.44−1.34 (m, 4H), 1.27–1.13 (m, 8H).

^13^C NMR (101 MHz, DMSO) δ 172.43, 171.76, 169.95, 74.84, 47.55, 47.25, 46.21, 40.62, 40.42, 40.21, 40.00, 39.79, 39.58, 39.37, 38.88, 38.46, 30.37, 29.29, 28.04, 26.50, 23.96, 21.72, 20.82, 16.17.

**MS (ESI** ^**+**^ **):** *m*/*z* calculated for C_27_H_52_N_7_O_10_ [M + H]^+^: 634.378; found: 634.377; calculation for C_27_H_51_N_7_NaO_10_ [M + Na]^+^: 656.360; found: 656.359.

### Optimization, Stability, and Functionality Assays

Our cell radiolabeling protocol consists of three steps (Figure 1): 1) oxidation of cell surface sialic acids to aldehydes using sodium periodate (NaIO_4_), 2) covalent conjugation of AOD to these newly formed aldehydes via oxime ligation, with aniline used as a nucleophilic catalyst to enhance reaction efficiency, and 3) radiolabeling of cell-bound DFO with ^89^Zr.

We first optimized the oxidation and conjugation steps, by varying time, pH, and reagent concentration to determine suitable reaction conditions that maximize cell viability while ensuring effective AOD incorporation. U937 cells were employed for method development and optimization. Oxidation conditions evaluate the effects of time, pH, and reagent concentration. U937 cells were incubated with different concentrations of NaIO_4_ (0.1–10 mM) in PBS at pH values ranging from 6.0 to 7.4 for up to 2 h. 20-μL cell aliquots were isolated at 30, 60, and 120 min of incubation and cell viability was measured by trypan blue staining.

Similarly, the conjugation step was optimized first for cell viability by varying both the concentration of AOD (50–500 μM) in the presence (*N* = 3) or absence (*N* = 1) of the catalyst aniline (10 mM). U937 cells were incubated under these different conditions for up to 2 h, with 20-μL aliquots collected at 30, 60, 90, and 120 min to assess viability using trypan blue staining. Next, to determine if the catalyst aniline is necessary for efficient radiolabeling, we performed the oxidation step using the optimized conditions (1 mM NaIO_4_ in pH 7.4 PBS at room temperature for 20 min), subsequently conjugated the cells with AOD (200 μM) in the presence or absence of aniline (10 mM), radiolabeled the U937 cells, and assessed radiochemical incorporation, cell viability, and radiochemical purity across each condition (*N* = 3).

Previous reagents and cellular debris were removed between each reaction step by multiple centrifugation steps, with spin speed and duration determined by cell characteristics (size, density, 200–600*g*, 5 min).

### Cell Radiolabeling Methodology

AOD-conjugated cells were radiolabeled with ^89^Zr (0.5–1 mCi per million cells) in pH 7.4 PBS for 40 min. Radiolabeling reagents and free ^89^Zr were removed after the radiolabeling through multiple centrifugation steps. Cell viability was measured at all steps via trypan blue staining. For all radiolabeling studies, ^89^Zr-oxalate was pH adjusted to pH ∼7.4 using 1 M HEPES buffer and 1 M NaHCO_3_.

Radiochemical yield and purity were determined via radiosensitive instant thin-layer chromatography (iTLC). iTLC was performed by suspending radiolabeled cells in either PBS or complete cell culture media (1 μL). These suspensions were spotted onto silica-coated aluminum sheets (Millipore Corporation, Burlington, MA), allowed to dry, and developed using ethylenediaminetetraacetic acid (EDTA, 50 mM) as the mobile phase. Lanes were scanned using a Cyclone Plus Storage Phosphor System (PerkinElmer, Waltham, MA) and quantified with OptiQuant software (PerkinElmer, Waltham, MA). Results were reported as the relative total area percentage, with background correction applied.

With the three-step direct radiolabeling protocol established and optimized, five cell types were radiolabeled to evaluate the method’s generalizability. These were human U937 cells, PBMCs, and Jurkat T cells; and NHP neutrophils and T cells. For each cell type, three key parameters were assessed: radiochemical purity, measured by iTLC; radioactivity incorporation, quantified using a well counter (Capintec CRC-55tW, Florham Park, NJ); and cell viability, determined via trypan blue staining using an Accuris QuadCount (Edison, NJ).

### Radiochemical Stability

Radiochemical purity and cell viability were measured across 48 h to evaluate the stability of the labeling. Following the final purification after radiolabeling, [^89^Zr]­Zr–U937 cells (90–115 μCi, 2–3 × 10^6^ cells, *N* = 4) were resuspended in 5 mL of complete cell culture media (RPMI 1640 + 10% Fetal Bovine Serum) and stored at 37 °C in 5% CO_2_. Samples were collected at 2, 18, 24, 36, and 48 h post-radiolabeling and assayed for radiochemical stability using iTLC and cell viability using trypan blue staining.

### Phagocytosis Assay

To evaluate whether radiolabeling affected the phagocytic ability of U937 cells, [^89^Zr]­Zr–U937 cells and unlabeled U937 cells (3–4 × 10^6^ cells, 100 μL) were incubated with latex beads conjugated to rabbit IgG-FITC (1:100 dilution, Cayman Chemical, Ann Arbor, MI) for 30 min. After incubation, cells were centrifuged (400*g*, 5 min) and resuspended in the provided assay buffer. Samples were analyzed using an Attune NXT flow cytometer (Thermo Fisher Scientific) calibrated for FITC wavelengths (excitation/emission: 485 nm/535 nm), with a minimum of 10,000 live events collected per sample to ensure data reliability. Flow cytometry data were processed and analyzed using FlowJo software (Ashland, OR).

### In Vivo PET/CT and Biodistribution

To evaluate the in vivo stability of the ^89^Zr radiolabel and assess the biodistribution of different cell types, radiolabeled cells were intravenously administered to NSG mice. For comparison, PET/CT imaging of three mice injected with [^89^Zr]­Zr-DFO was also performed. Micro-PET/CT imaging was performed using a Siemens Inveon scanner (Malvern, PA) at 4–5 time points over a one-week period, ranging from 2 to 192 h post-injection. PET scans were reconstructed with a three-dimensional ordered subsets expectation maximization algorithm (OSEM-3D) and corrected for attenuation and decay, without application of scatter correction.

Following the final PET/CT scan, ex vivo biodistribution studies were conducted to corroborate the quantitative ROI analysis from the imaging data. Organs were harvested, radioactivity was measured using a gamma counter (Wizard2, PerkinElmer, Waltham, MA), and the results are provided (Figures S9–13, Table S7).

### Statistics

Data are presented as mean ± standard error. Student’s *t* tests were performed, with *p* < 0.05 considered statistically significant.

## Supplementary Material





## Notes

^1^H NMR spectra, ^13^C NMR spectra, mass spectra, and HPLC traces for Compound 1 and aminooxy-DFO; [^89^Zr]­Zr-Jurkat cell maximum intensity projections and region of interest analysis; [^89^Zr]­Zr-DFO maximum intensity projections, region of interest analysis, and instant thin layer chromatography data; ex vivo biodistribution data from in vivo tracking studies; raw data for ROI quantification and ex vivo biodistribution (PDF)
Molecular formula strings (CSV)

## Abbreviations

AOD: Aminooxy-DFO
CAR: Chimeric Antigen Receptor
DFO: Deferoxamine
GVHD: Graft Versus Host Disease
HPC: Hematopoietic Progenitor Cell
iPSC: Induced Pluripotent Stem Cell
iTLC: Instant Thin Layer Chromatography
MIP: Maximum Intensity Projection
MNC: Mononuclear Cell
MSC: Mesenchymal Stromal Cell
NHP: Nonhuman Primate
NSG: NOD-SCID-γc^–/–^
OSEM-3D: Ordered Subsets Expectation Maximization
PBMC: Peripheral Blood Mononuclear Cell
PET/CT: Positron Emission Tomography/Computed Tomography
ROI: Region of Interest
SPECT: Single Photon Emission Computed Tomography
TCR: T Cell Receptor
TIL: Tumor Infiltrating Lymphocyte.

## References

[1] Tang J., Hubbard-Lucey V. M., Pearce L., O’Donnell-Tormey J., Shalabi A. (2018). The Global
Landscape of Cancer Cell Therapy. Nat. Rev.
Drug Discovery.

[2] Guedan S., Ruella M., June C. H. (2019). Emerging
Cellular Therapies for Cancer. Annu. Rev. Immunol..

[3] Neelapu S. S., Locke F. L., Bartlett N. L., Lekakis L. J., Miklos D. B., Jacobson C. A., Braunschweig I., Oluwole O. O., Siddiqi T., Lin Y., Timmerman J. M., Stiff P. J., Friedberg J. W., Flinn I. W., Goy A., Hill B. T., Smith M. R., Deol A., Farooq U., McSweeney P., Munoz J., Avivi I., Castro J. E., Westin J. R., Chavez J. C., Ghobadi A., Komanduri K. V., Levy R., Jacobsen E. D., Witzig T. E., Reagan P., Bot A., Rossi J., Navale L., Jiang Y., Aycock J., Elias M., Chang D., Wiezorek J., Go W. Y. (2017). Axicabtagene
Ciloleucel CAR T-Cell Therapy in Refractory Large B-Cell Lymphoma. N. Engl. J. Med..

[4] Raje N., Berdeja J., Lin Y., Siegel D., Jagannath S., Madduri D., Liedtke M., Rosenblatt J., Maus M. V., Turka A., Lam L.-P., Morgan R. A., Friedman K., Massaro M., Wang J., Russotti G., Yang Z., Campbell T., Hege K., Petrocca F., Quigley M. T., Munshi N., Kochenderfer J. N. (2019). Anti-BCMA
CAR T-Cell Therapy Bb2121 in Relapsed or Refractory Multiple Myeloma. N. Engl. J. Med..

[5] Rosenberg S. A., Yang J. C., Sherry R. M., Kammula U. S., Hughes M. S., Phan G. Q., Citrin D. E., Restifo N. P., Robbins P. F., Wunderlich J. R., Morton K. E., Laurencot C. M., Steinberg S. M., White D. E., Dudley M. E. (2011). Durable Complete
Responses in Heavily Pretreated Patients with Metastatic Melanoma
Using T-Cell Transfer Immunotherapy. Clin. Cancer
Res..

[6] Fry T. J., Shah N. N., Orentas R. J., Stetler-Stevenson M., Yuan C. M., Ramakrishna S., Wolters P., Martin S., Delbrook C., Yates B., Shalabi H., Fountaine T. J., Shern J. F., Majzner R. G., Stroncek D. F., Sabatino M., Feng Y., Dimitrov D. S., Zhang L., Nguyen S., Qin H., Dropulic B., Lee D. W., Mackall C. L. (2018). CD22-Targeted CAR
T Cells Induce Remission in B-ALL That Is Naive or Resistant to CD19-Targeted
CAR Immunotherapy. Nat. Med..

[7] US Food and Drug Administration; HEMACORD (Hematopoietic Progenitor Cells, Cord Blood), 2011.

[8] Eapen M., Rubinstein P., Zhang M.-J., Stevens C., Kurtzberg J., Scaradavou A., Loberiza F. R., Champlin R. E., Klein J. P., Horowitz M. M., Wagner J. E. (2007). Outcomes of Transplantation
of Unrelated
Donor Umbilical Cord Blood and Bone Marrow in Children with Acute
Leukaemia: A Comparison Study. The Lancet.

[9] Kurtzberg J., Prasad V. K., Carter S. L., Wagner J. E., Baxter-Lowe L. A., Wall D., Kapoor N., Guinan E. C., Feig S. A., Wagner E. L., Kernan N. A. (2008). Results of the Cord Blood Transplantation
Study (COBLT): Clinical Outcomes of Unrelated Donor Umbilical Cord
Blood Transplantation in Pediatric Patients with Hematologic Malignancies. Blood.

[10] Ikeda K., Ohto H., Okuyama Y., Yamada-Fujiwara M., Kanamori H., Fujiwara S., Muroi K., Mori T., Kasama K., Iseki T., Nagamura-Inoue T., Fujii N., Ashida T., Kameda K., Kanda J., Hirose A., Takahashi T., Nagai K., Minakawa K., Tanosaki R. (2018). Adverse Events Associated With Infusion of Hematopoietic
Stem Cell Products: A Prospective and Multicenter Surveillance Study. Transfus Med. Rev..

[11] Etra A., Ferrara J. L. M., Levine J. E. (2025). Remestemcel-L-Rknd
(Ryoncil): The
First Approved Cellular Therapy for Steroid-Refractory Acute GVHD. Blood.

[12] Smith S. R., Munavalli G., Weiss R., Maslowski J. M., Hennegan K. P., Novak J. M. A. M. D.-B. (2012). Placebo-Controlled Trial of Autologous
Fibroblast Therapy for the Treatment of Nasolabial Fold Wrinkles. Dermatol. Surg..

[13] Brittberg M., Recker D., Ilgenfritz J., Saris D. B. F., on
behalf of the SUMMIT Extension Study Group (2018). Matrix-Applied Characterized Autologous Cultured Chondrocytes
Versus Microfracture: Five-Year Follow-up of a Prospective Randomized
Trial. Am. J. Sports Med..

[14] Lulla P. D., Brenner M. (2023). Emerging Challenges to Cellular Therapy
of Cancer. Cancer J..

[15] James M. L., Gambhir S. S. (2012). A Molecular Imaging
Primer: Modalities, Imaging Agents,
and Applications. Physiol. Rev..

[16] Bulte J. W. M., Daldrup-Link H. E. (2018). Clinical
Tracking of Cell Transfer
and Cell Transplantation: Trials and Tribulations. Radiology.

[17] Ashmore-Harris C., Iafrate M., Saleem A., Fruhwirth G. O. (2020). Non-Invasive
Reporter Gene Imaging of Cell Therapies, Including T Cells and Stem
Cells. Mol. Ther..

[18] Ritchie D., Mileshkin L., Wall D., Bartholeyns J., Thompson M., Coverdale J., Lau E., Wong J., Eu P., Hicks R. J., Prince H. M. (2006). In Vivo
Tracking of Macrophage Activated
Killer Cells to Sites of Metastatic Ovarian Carcinoma. Cancer Immunol. Immunother..

[19] Jeong H. J., Yoo R. J., Kim J. K., Kim M. H., Park S. H., Kim H., Lim J. W., Do S. H., Lee K. C., Lee Y. J., Kim D. W. (2019). Macrophage Cell
Tracking PET Imaging Using Mesoporous
Silica Nanoparticles via in Vivo Bioorthogonal F-18 Labeling. Biomaterials.

[20] Sato N., Stringaris K., Davidson-Moncada J. K., Reger R., Adler S. S., Dunbar C., Choyke P. L., Childs R. W. (2020). *In Vivo* Tracking
of Adoptively Transferred Natural Killer Cells in Rhesus
Macaques Using 89Zirconium-Oxine Cell Labeling and PET Imaging. Clin. Cancer Res..

[21] Bansal A., Pandey M. K., Demirhan Y. E., Nesbitt J. J., Crespo-Diaz R. J., Terzic A., Behfar A., DeGrado T. R. (2015). Novel 89Zr Cell
Labeling Approach for PET-Based Cell Trafficking Studies. EJNMMI Res..

[22] Weist M. R., Starr R., Aguilar B., Chea J., Miles J. K., Poku E., Gerdts E., Yang X., Priceman S. J., Forman S. J., Colcher D., Brown C. E., Shively J. E. (2018). PET of
Adoptively Transferred Chimeric Antigen Receptor T Cells with^89^ Zr-Oxine. J. Nucl. Med..

[23] Asiedu K. O., Koyasu S., Szajek L. P., Choyke P. L., Sato N. (2017). Bone Marrow
Cell Trafficking Analyzed by 89Zr-Oxine Positron Emission Tomography
in a Murine Transplantation Model. Clin. Cancer
Res..

[24] Stanley, P. ; Wuhrer, M. ; Lauc, G. ; Stowell, S. R. ; Cummings, R. D. Structures Common to Different Glycans. In Essentials of Glycobiology; Varki, A. ; Cummings, R. D. ; Esko, J. D. ; Stanley, P. ; Hart, G. W. ; Aebi, M. ; Mohnen, D. ; Kinoshita, T. ; Packer, N. H. ; Prestegard, J. H. ; Schnaar, R. L. ; Cold Spring Harbor Laboratory Press; Cold Spring Harbor, NY, 2022.

[25] Cheng B., Tang Q., Zhang C., Chen X. (2021). Glycan Labeling and
Analysis in Cells and In Vivo. Annu. Rev. Anal
Chem..

[26] Zeng Y., Ramya T. N. C., Dirksen A., Dawson P. E., Paulson J. C. (2009). High-Efficiency
Labeling of Sialylated Glycoproteins on Living Cells. Nat. Methods.

[27] Sarkar B., Jayaraman N. (2020). Glycoconjugations of Biomolecules by Chemical Methods. Front. Chem..

[28] Shivatare S. S., Shivatare V. S., Wong C.-H. (2022). Glycoconjugates: Synthesis, Functional
Studies, and Therapeutic Developments. Chem.
Rev..

[29] Varki N. M., Varki A. (2007). Diversity in Cell Surface Sialic
Acid Presentations: Implications
for Biology and Disease. Lab. Invest..

[30] Abou D. S., Ku T., Smith-Jones P. M. (2011). In Vivo Biodistribution and Accumulation
of 89Zr in Mice. Nucl. Med. Biol..

[31] Tekin V., Archer N. E., Fernandez S. R., Houson H. A., Bartels J. L., Lapi S. E. (2025). Homotypic Targeting
of [89Zr]­Zr-Oxine Labeled PC3 and
4T1 Cells in Tumor-Bearing Mice. Pharmaceutics.

[32] Koyasu S., Minor H. A., Asiedu K. O., Choyke P. L., Sato N. (2025). Zirconium-89-Oxine
Cell Tracking by PET Reveals Preferential Monocyte Recruitment to
Cancer and Inflammation over Macrophages. Pharmaceuticals.

[33] Epstein J. S., Grobman L., Ganz W. I., Goodwin W. J., Lizak M., Dewanjee M. K. (1992). Indium 111–Labeled
Leukocyte Scintigraphy in
Evaluating Head and Neck Infections. Ann. Otol.
Rhinol. Laryngol..

[34] Minoja G., Chiaranda M., Fachinetti A., Raso M., Dominioni L., Torre D., De Palma D. (1996). The Clinical Use of 99m-Tc-Labeled
WBC Scintigraphy in Critically Ill Surgical and Trauma Patients with
Occult Sepsis. Intensive Care Med..

[35] Gawne P. J., Man F., Blower P. J., de Rosales T. M. D. (2022). Direct Cell Radiolabeling for in
Vivo Cell Tracking with PET and SPECT Imaging. Chem. Rev..

[36] Helfer B. M., Ponomarev V., Patrick P. S., Blower P. J., Feitel A., Fruhwirth G. O., Jackman S., Pereira Mouriès L., Park M. V. D. Z., Srinivas M., Stuckey D. J., Thu M. S., Van Den Hoorn T., Herberts C. A., Shingleton W. D. (2021). Options
for Imaging Cellular Therapeutics in Vivo: A Multi-Stakeholder Perspective. Cytotherapy.

[37] Kim M. H., Lee Y. J., Kang J. H. (2016). Stem Cell Monitoring
with a Direct
or Indirect Labeling Method. Nucl. Med. Mol.
Imaging..

[38] Lee S. H., Soh H., Chung J. H., Cho E. H., Lee S. J., Ju J.-M., Sheen J. H., Kim H., Oh S. J., Lee S.-J., Chung J., Choi K., Kim S.-Y., Ryu J.-S. (2020). Feasibility
of Real-Time in Vivo 89Zr-DFO-Labeled CAR T-Cell Trafficking Using
PET Imaging. PLoS One.

[39] Golke T., Mucher P., Schmidt P., Radakovics A., Repl M., Hofer P., Perkmann T., Fondi M., Schmetterer K. G., Haslacher H. (2022). Delays during
PBMC Isolation Have
a Moderate Effect on Yield, but Severly Compromise Cell Viability. Clin. Chem. Lab. Med. CCLM.

[40] Grievink H. W., Luisman T., Kluft C., Moerland M., Malone K. E. (2016). Comparison
of Three Isolation Techniques for Human Peripheral Blood Mononuclear
Cells: Cell Recovery and Viability, Population Composition, and Cell
Functionality. Biopreservation Biobanking.

[41] Kalia J., Raines R. T. (2008). Hydrolytic Stability of Hydrazones and Oximes. Angew. Chem., Int. Ed..

[42] Bubenshchikov V., Makichyan A., Larenkov A. (2022). The Impact of Zirconium-89 Solution
Formulation on the Efficiency of [^89^ Zr]­Zr-deferoxamine
Synthesis. J. Label. Compd. Radiopharm..

[43] Bellotti D., Remelli M. D. B. (2021). A Natural, Excellent
and Versatile Metal Chelator. Molecules.

[44] Charoenphun P., Meszaros L. K., Chuamsaamarkkee K., Sharif-Paghaleh E., Ballinger J. R., Ferris T. J., Went M. J., Mullen G. E. D., Blower P. J. (2015). [89Zr]­Oxinate4 for Long-Term in Vivo
Cell Tracking
by Positron Emission Tomography. Eur. J. Nucl.
Med. Mol. Imaging..

[45] Brenner W., Aicher A., Eckey T., Massoudi S., Zuhayra M., Koehl U., Heeschen C., Kampen W. U., Zeiher A. M., Dimmeler S. (2004). 111In-Labeled CD34+
Hematopoietic Progenitor
Cells in a Rat Myocardial Infarction Model. J. Nucl. Med..

[46] Kuyama J., McCormack A., George A. J. T., Heelan B. T., Osman S., Batchelor J. R., Peters A. M. (1997). Indium-111 Labelled Lymphocytes:
Isotope Distribution and Cell Division. Eur.
J. Nucl. Med..

[47] Klaver Y., Kunert A., Sleijfer S., Debets R., Lamers C. H. (2015). Adoptive
T-Cell Therapy: A Need for Standard Immune Monitoring. Immunotherapy.

[48] Ploch W., Sadowski K., Olejarz W., Basak G. W. (2024). Advancement and
Challenges in Monitoring of CAR-T Cell Therapy: A Comprehensive Review
of Parameters and Markers in Hematological Malignancies. Cancers.

[49] Bhattacharya A., Kochhar R., Sharma S., Ray P., Kalra N., Khandelwal N., Mittal B. R. (2014). PET/CT with^18^ F-FDG–Labeled
Autologous Leukocytes for the Diagnosis of Infected Fluid Collections
in Acute Pancreatitis. J. Nucl. Med..

[50] Wang X., Rosol M., Ge S., Peterson D., McNamara G., Pollack H., Kohn D. B., Nelson M. D., Crooks G. M. (2003). Dynamic
Tracking of Human Hematopoietic Stem Cell Engraftment Using in Vivo
Bioluminescence Imaging. Blood.

[51] Dobrenkov K., Olszewska M., Likar Y., Shenker L., Gunset G., Cai S., Pillarsetty N., Hricak H., Sadelain M., Ponomarev V. (2008). Monitoring
the Efficacy of Adoptively Transferred Prostate Cancer–Targeted
Human T Lymphocytes with PET and Bioluminescence Imaging. J. Nucl. Med..

[52] Silva Z., Rabaça J. A., Luz V., Lourenço R. A., Salio M., Oliveira A. C., Bule P., Springer S., Videira P. A. (2025). New Insights into
the Immunomodulatory Potential of
Sialic Acid on Monocyte-Derived Dendritic Cells. Cancer Immunol., Immunother..

[53] Schmidt M., Linder A. T., Korn M., Schellenberg N., Meyer S. J., Nimmerjahn F., Werner A., Abeln M., Gerardy-Schahn R., Münster-Kühnel A. K., Nitschke L. (2024). Sialic Acids on T Cells Are Crucial for Their Maintenance
and Survival. Front. Immunol..

[54] D’Souza S. S., Maufort J., Kumar A., Zhang J., Smuga-Otto K., Thomson J. A., Slukvin I. I. (2016). GSK3β
Inhibition Promotes Efficient
Myeloid and Lymphoid Hematopoiesis from Non-Human Primate-Induced
Pluripotent Stem Cells. Stem Cell Rep..

[55] Kumar A., D’Souza S., Uenishi G., Park M., Lee J., Slukvin I. (2020). Generation of T Cells from Human and Nonhuman Primate
Pluripotent Stem Cells. BIO-Protoc..

