# Supplementary material for: Biocompatible Glycoconjugation Enables Sensitive In Vivo Cell Tracking by PET/CT
Source: J Med Chem. 2026 Jun 24;69(13):15570–80. doi: 10.1021/acs.jmedchem.6c00538 (PMC13370869; doi:10.1021/acs.jmedchem.6c00538)
Supplement: Supplementary file 1 [file jm6c00538_si_001.pdf]

Supporting Information

Biocompatible Glycoconjugation Enables Sensitive *In Vivo* Cell Tracking by PET/CT

Nathan Clemons,[a] Anna S. Thickens,[a] Liudmila Lambert Lepesevich,[a] Zachary T. Rosenkrans,[a,e] Victor Santoro-Fernandes,[a] Anatoly N. Pinchuk,[a] Eduardo Aluicio-Sarduy,[a] Jason C. Mixdorf,[a] Saritha S. D'Souza,[b] Johnathan Caldon,[b] John Kink,[g] Matthew H. Forsberg,[g] Peiman Hematti,[f] Jonathan W. Engle,[a,e] Igor Slukvin,[b,c,d,e] Christian M. Capitini,[g,e] Reinier Hernandez[a,e*].

[a]Departments of Medical Physics and Radiology, University of Wisconsin School of Medicine and Public Health, 1111 Highland Ave., Madison, WI 53705, USA

[b]Wisconsin National Primate Research Center, University of Wisconsin-Madison, 1220 Capitol Court, Madison, WI 53792, USA

[c]Department of Pathology and Laboratory Medicine, University of Wisconsin School of Medicine and Public Health, 1685 Highland Ave., Madison, WI 53705, USA

[d]Department of Cell and Regenerative Biology, University of Wisconsin School of Medicine and Public Health, 1111 Highland Ave., Madison, WI 53705, USA

[e]Carbone Cancer Center, University of Wisconsin School of Medicine and Public Health, 600 Highland Ave., Madison, WI 53792, USA

[f]Division of Hematology/Oncology, Medical College of Wisconsin, 9200 W. Wisconsin Ave., Milwaukee, WI 53226, USA

[g]Department of Pediatrics, University of Wisconsin School of Medicine and Public Health, 600 Highland Ave., Madison, WI 53792, USA

*To whom correspondence should be addressed. Address: Wisconsin Institutes for Medical Research, 1111 Highland Ave., Madison, WI 53705. Phone: 608-890-2771; Email: hernandez6@wisc.edu

Contents of Supporting Information

^{1}H-NMR Spectra for Compound **1** and AOD	S3-S4
^{13}C-NMR Spectra for Compound **1** and AOD	S5-S6
Mass Spectra for Compound **1** and AOD	S7-S8
HPLC Traces for Compound **1** and AOD	S9-S10
[^{89}Zr]Zr-Jurkat Data	S11-S12
Ex Vivo Biodistribution Graphs	S13-S17
[^{89}Zr]Zr-DFO Data	S18-S20
Region of Interest (ROI) Quantification Data	S21-S26
Ex Vivo Biodistribution Data	S27

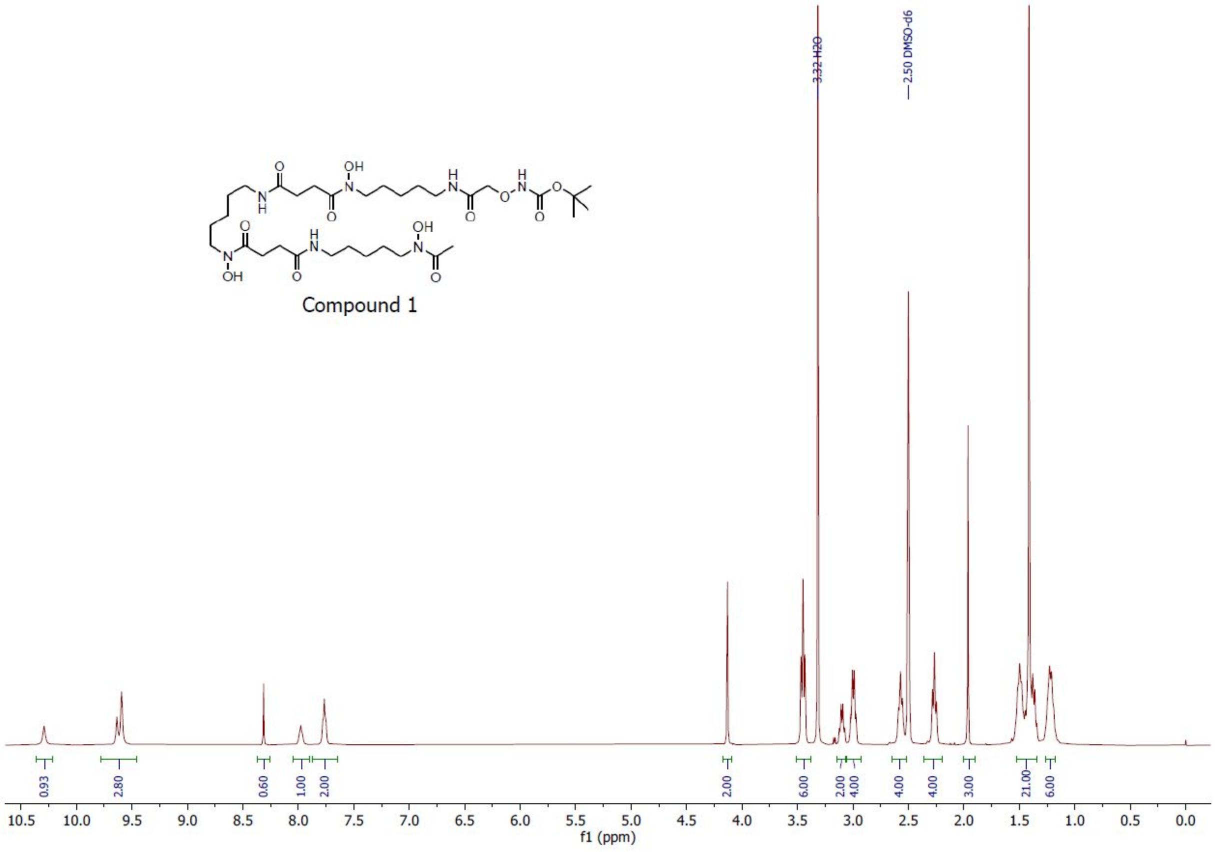

Figure S1. ^{1}H-NMR spectrum of compound **1**.

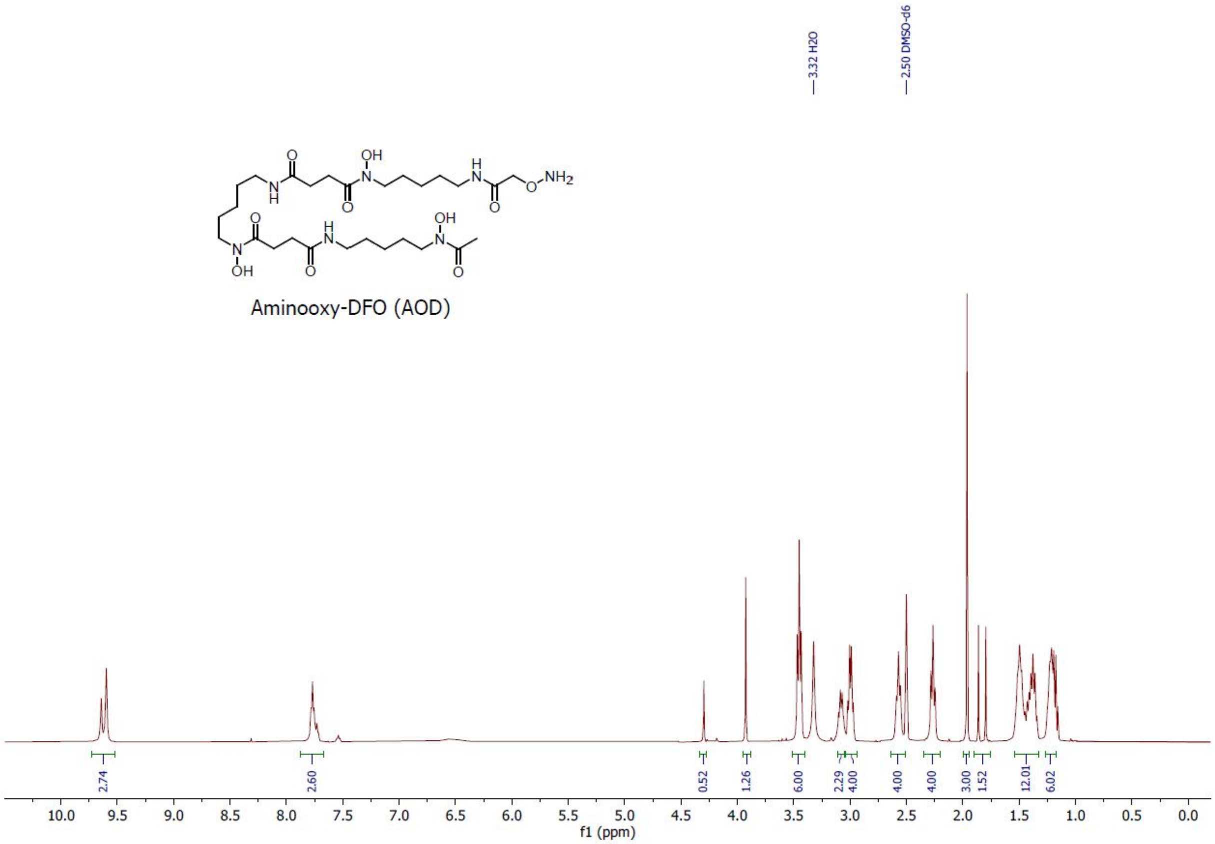

Figure S2. ^{1}H-NMR spectrum of Aminooxy-DFO (AOD).

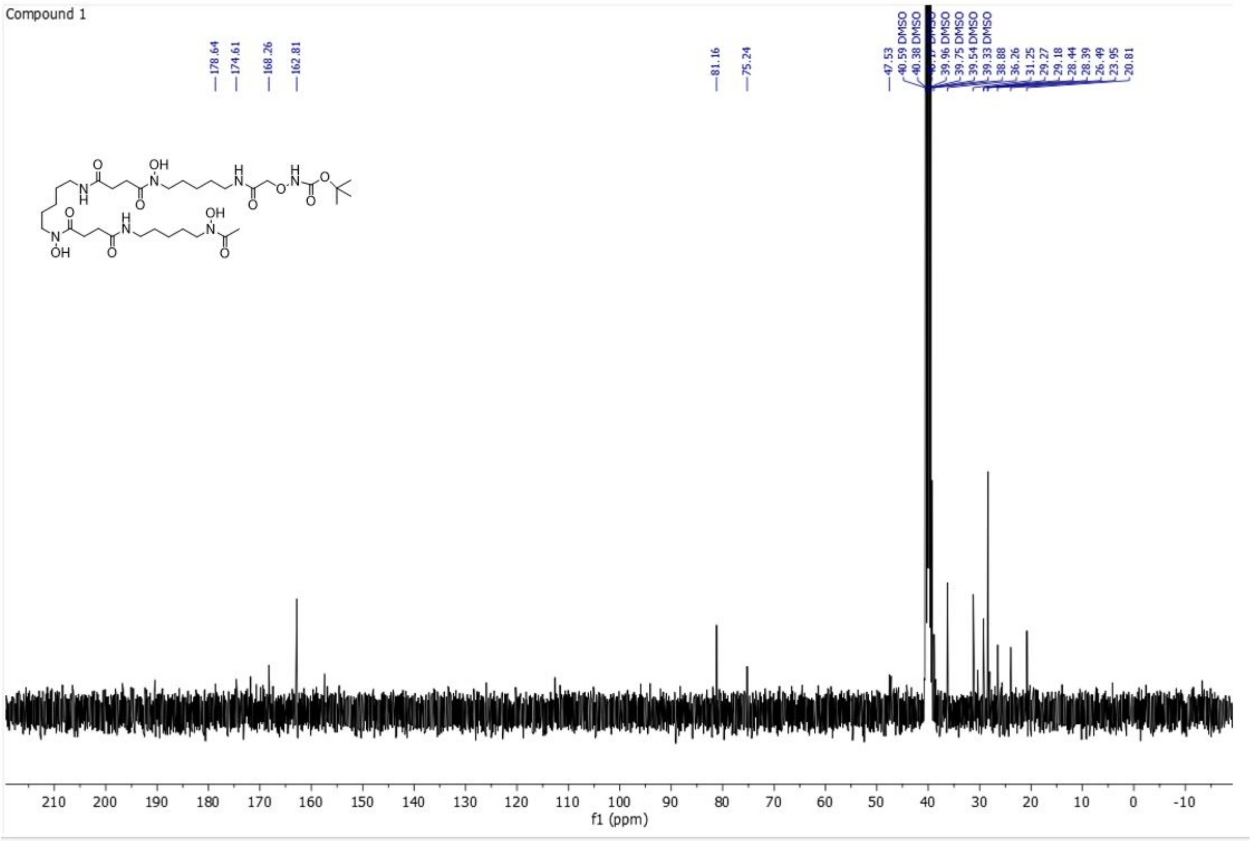

Figure S3. ^{13}C-NMR spectrum of compound **1**.

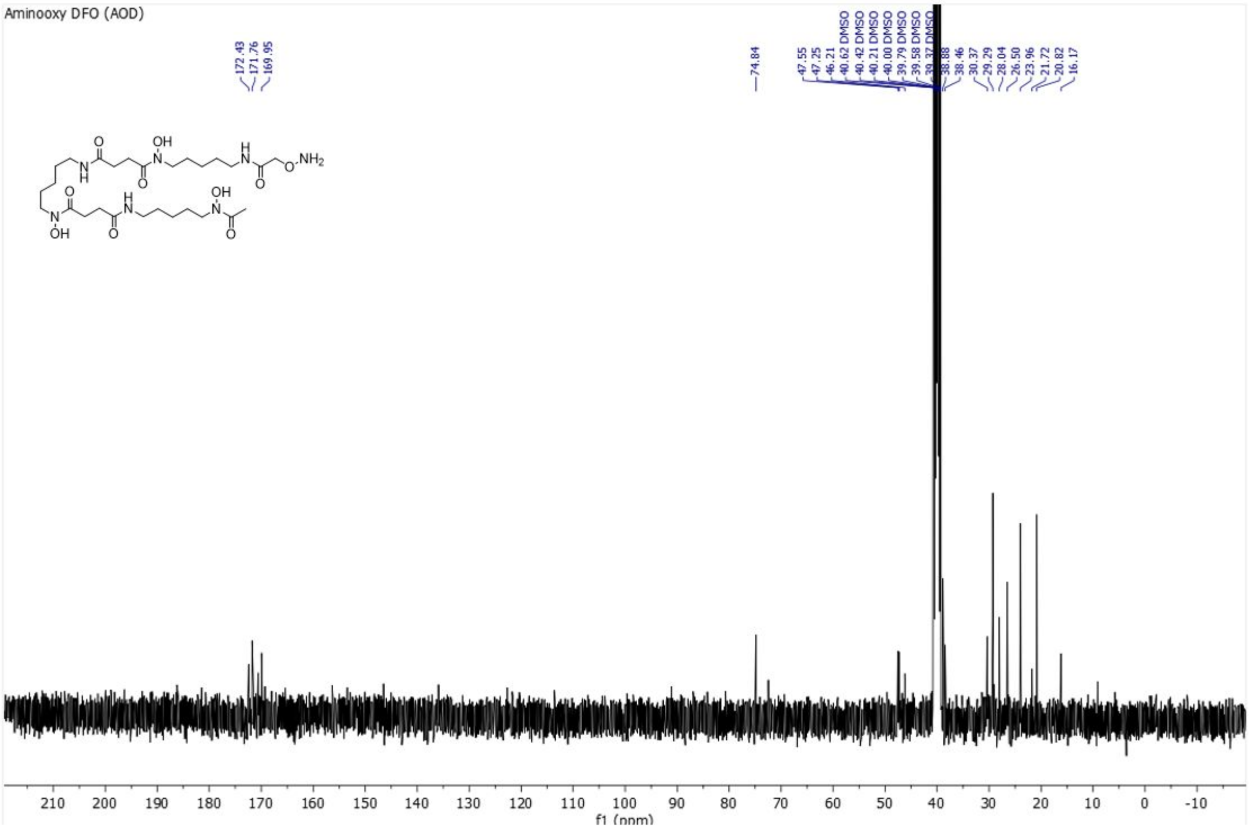

Figure S4. ^{13}C-NMR spectrum of Aminooxy-DFO (AOD).

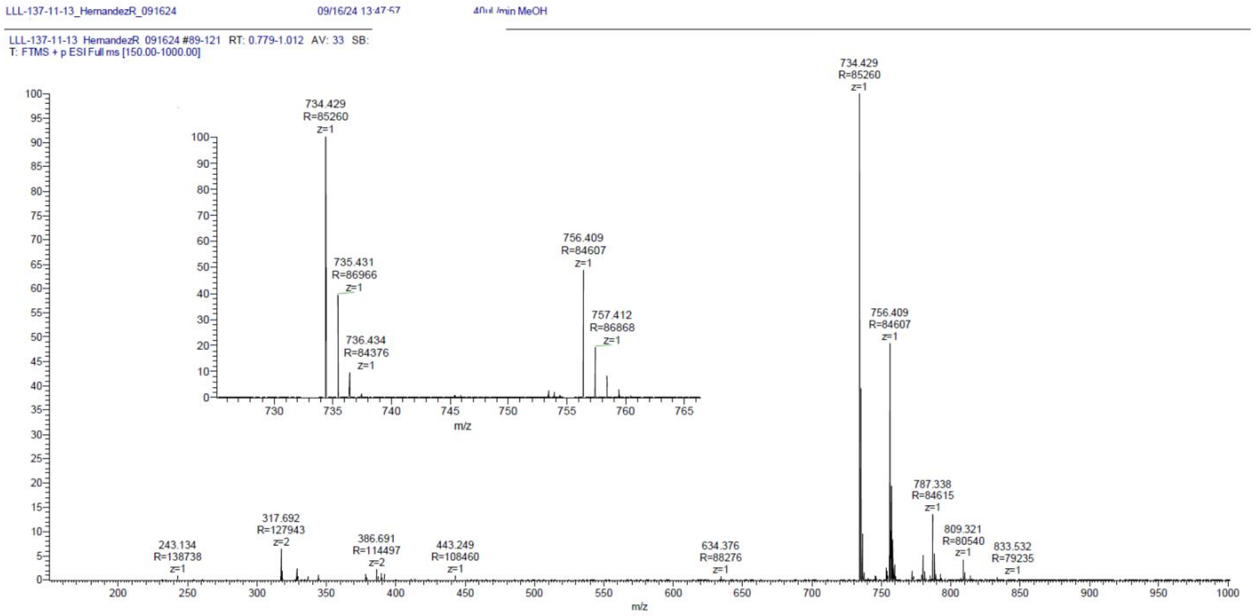

Figure S5. Mass spectrum of compound **1**.

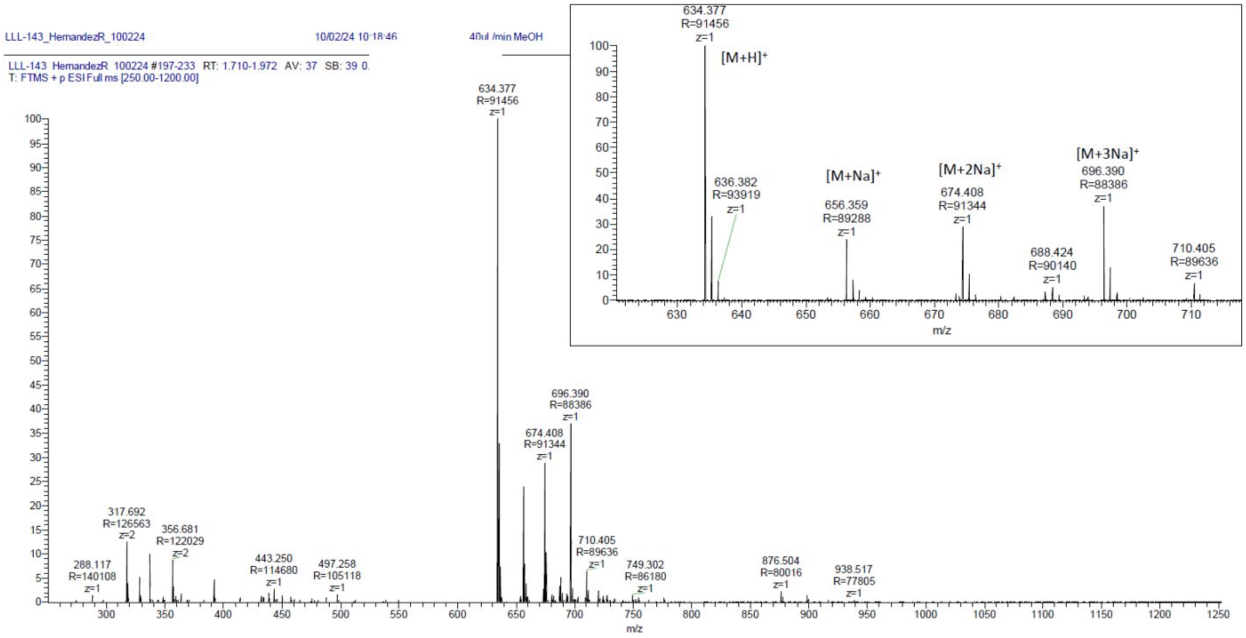

Figure S6. Mass spectrum of Aminooxy-DFO (AOD).

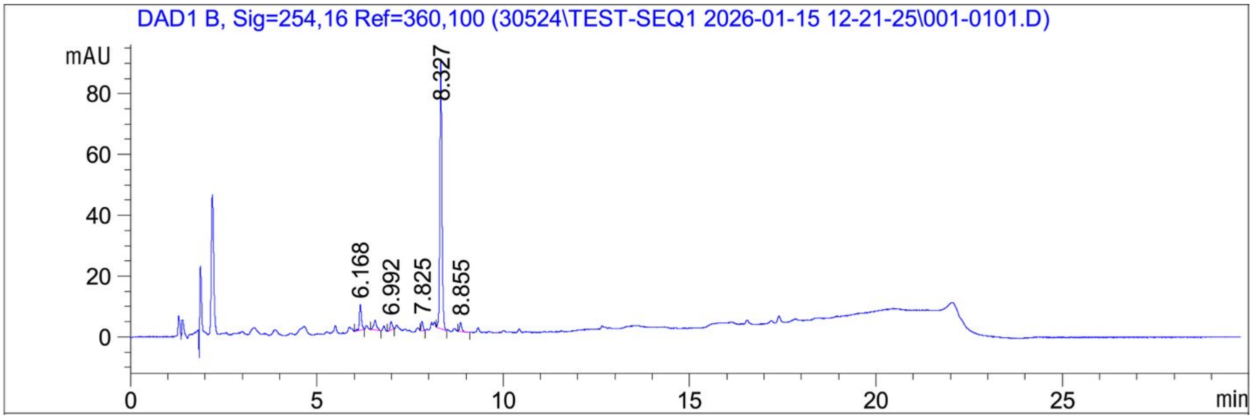

Figure S7. Analytical HPLC of AOD monitored at 254 nm. 20 µL of a 4.8 mg/mL solution of AOD was loaded onto a 250 x 4.6mm, 5 µm C18 Luna HPLC column (Phenomenex, Torrance, CA) and eluted with a linear 20-90% gradient of acetonitrile in water + 0.1% TFA at 1.18 mL/min for 30 minutes under ambient temperature. AOD eluted with a retention time of 8.327 minutes and a purity of 81% as determined by UV absorbance at 254 nm.

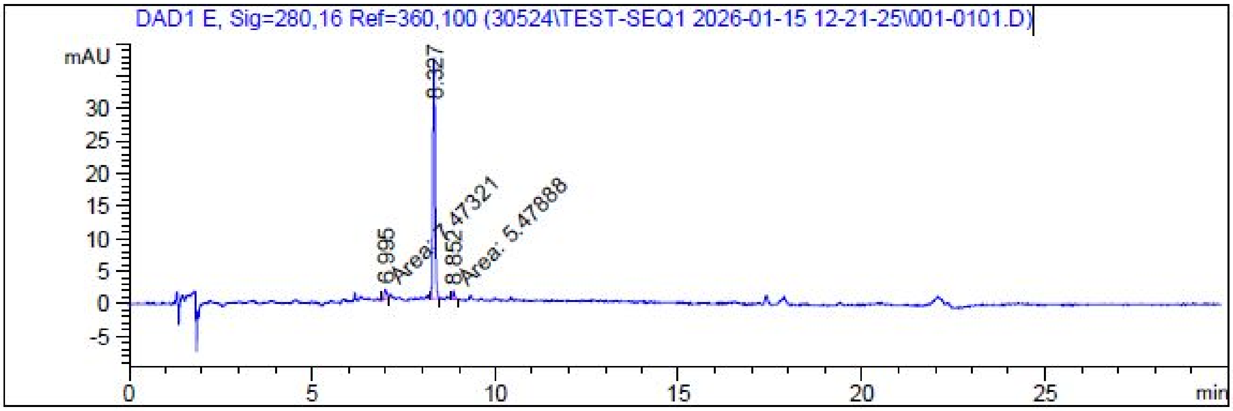

Figure S8. Analytical HPLC of AOD monitored at 280 nm. 20 µL of a 4.8 mg/mL solution of AOD was loaded onto a 250 x 4.6mm, 5 µm C18 Luna HPLC column (Phenomenex, Torrance, CA) and eluted with a linear 20-90% gradient of acetonitrile in water + 0.1% TFA at 1.18 mL/min for 30 minutes under ambient temperature. AOD eluted with a retention time of 8.327 minutes and a purity of 92% as determined by UV absorbance at 280 nm.

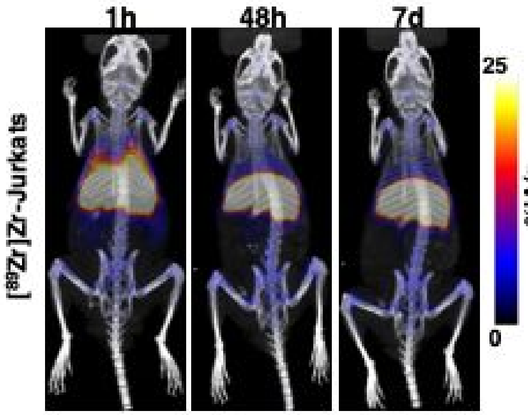

Figure S9. Maximum Intensity Projections (MIPs) PET/CT images of [^{89}Zr]Zr-Jurkat cells.

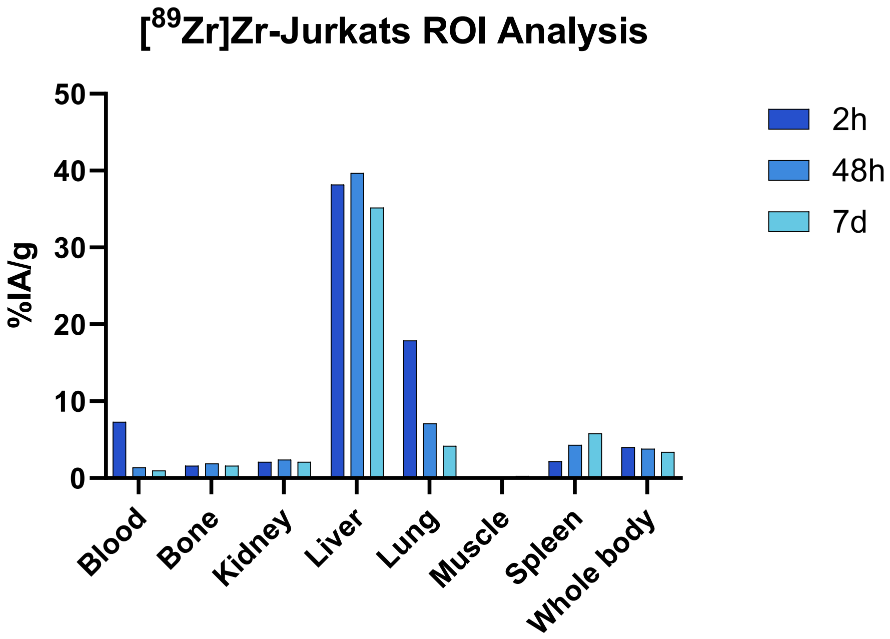

Figure S10. Region of Interest (ROI) analysis of [^{89}Zr]Zr-Jurkat cells PET/CT Imaging.

Ex vivo Biodistribution Studies

After the terminal PET/CT scan, mice were euthanized via inhalation of CO_2 followed by cervical dislocation. Organs of interest, including the heart, lungs, liver, spleen, kidney, stomach, intestine, bone, muscle, tail, skin, pancreas, and brain, were excised, weighed, and counted on a Wizard2 gamma counter (PerkinElmer, Waltham, MA). Ex vivo biodistribution was then calculated and reported as percent injected activity per gram of tissue (%IA/g) to corroborate the in vivo PET/CT imaging results.

The [^{89}Zr]Zr-U937 cells ex vivo biodistribution studies correlate with ROI analysis findings, with high liver (40.8 ± 8.9 %IA/g) and spleen (106.0 ± 27.4 %IA/g), and low bone (2.6 ± 0.8 %IA/g) and lung (3.6 ± 0.5 %IA/g) uptake at the 8 day timepoint (**Figure S11**). Ex vivo biodistribution studies of [^{89}Zr]Zr-PBMCs corroborated the ROI analysis, with high liver (53.1 %IA/g) and spleen (83.0 %IA/g) signal (**Figure S12**). Overall, trends observed in [^{89}Zr]Zr-PBMCs correlate strongly with [^{89}Zr]Zr-U937 cells, indicating the radiolabeling of [^{89}Zr]Zr-PBMCs is stable in vivo and capable of tracking [^{89}Zr]Zr-PBMCs over an 8 day period. [^{89}Zr]Zr-NHP Neutrophils also matched the ROI data, displaying high liver (65.14 ± 4.19 %IA/g) and spleen (85.54 ± 12.25 %IA/g) uptake (**Figure S13**). For [^{89}Zr]Zr-NHP -T cells, ex vivo biodistribution data again corroborated the PET/CT ROI analysis, with significant spleen (32.3 ± 8.5 %IA/g) and liver (52.6 ± 6.6 %IA/g) uptake after the 7d scan (**Figure S14**). Finally, [^{89}Zr]Zr-Jurkat cells matched their respective ROI analysis results, with high liver (56.48 %IA/g) and spleen (10.21 %IA/g) uptake. Notably, [^{89}Zr]Zr-Jurkat cells also showed heightened lung (15.74 %IA/g) and bone (5.25 %IA/g) uptake as compared to their 7d ROI analysis data (**Figure S15**).

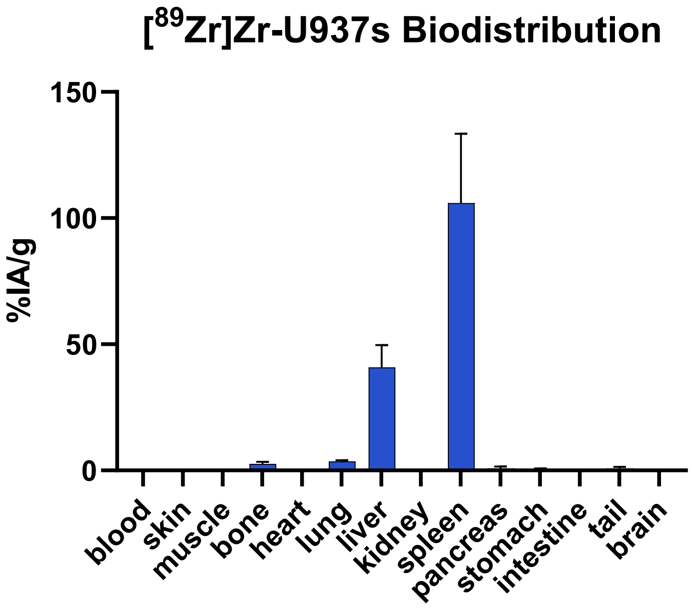

Figure S11. Ex Vivo Biodistribution of [^{89}Zr]Zr-U937 cells.

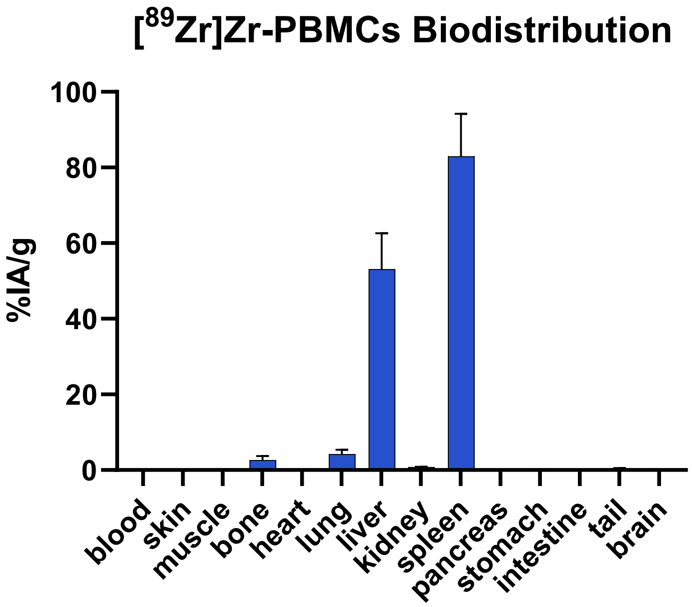

Figure S12. Ex Vivo Biodistribution of [^{89}Zr]Zr-PBMCs.

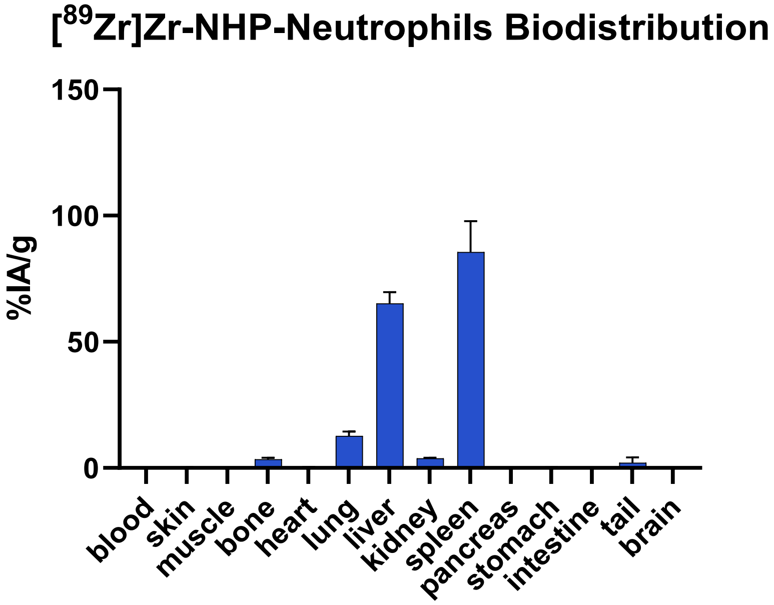

Figure S13. Ex Vivo Biodistribution of [^{89}Zr]Zr-NHP-Neutrophils.

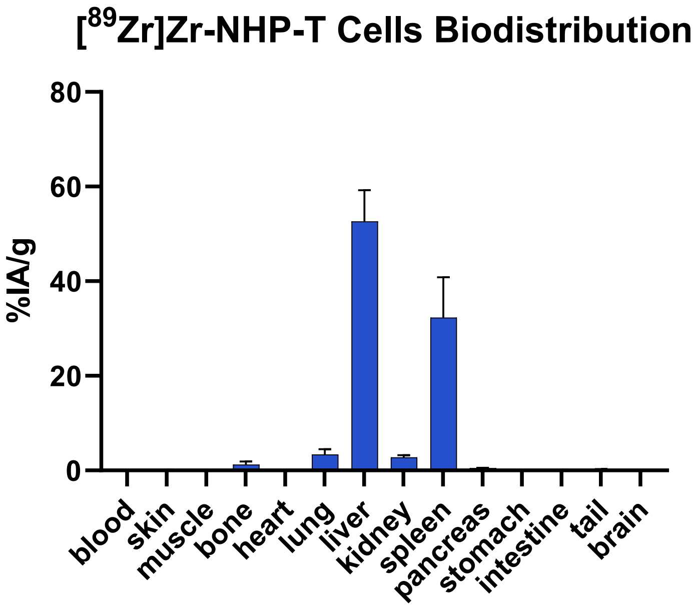

Figure S14. Ex Vivo Biodistribution of [^{89}Zr]Zr-NHP-T cells.

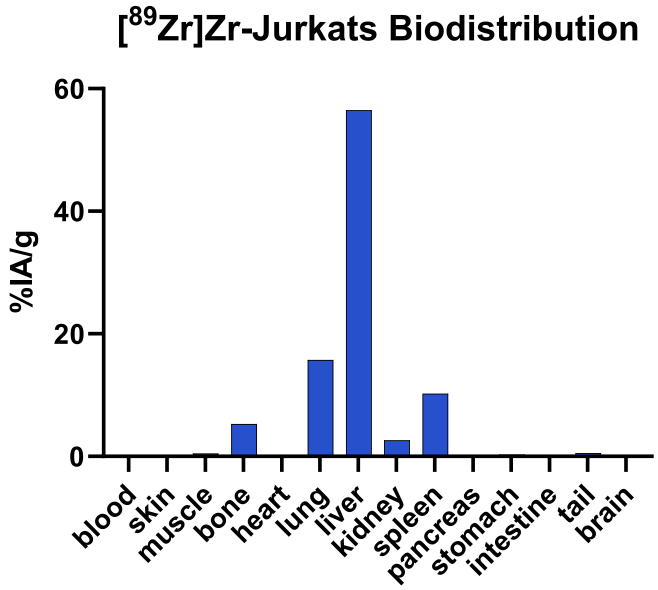

Figure S15. Ex Vivo Biodistribution of [^{89}Zr]Zr-Jurkat cells.

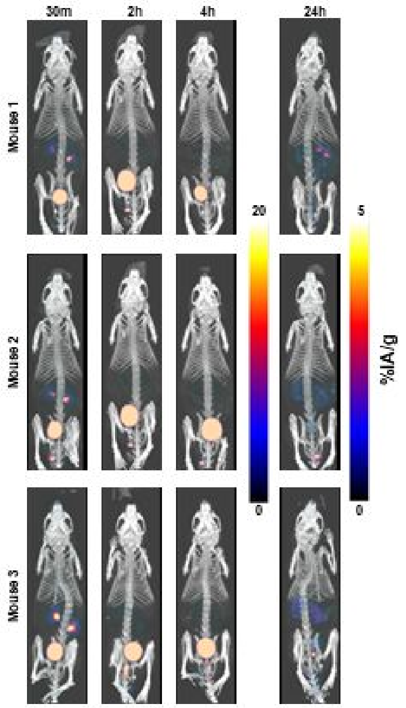

Figure S16. Maximum Intensity Projections (MIPs) of NSG mice intravenously injected with 108-112 µCi (4.0 – 4.1 MBq) [^{89}Zr]Zr-DFO and imaged using a Mediso NanoScan PET/CT (Mediso USA, Arlington, US) at 0.5, 2, 4, and 24h post-injection (N = 3).

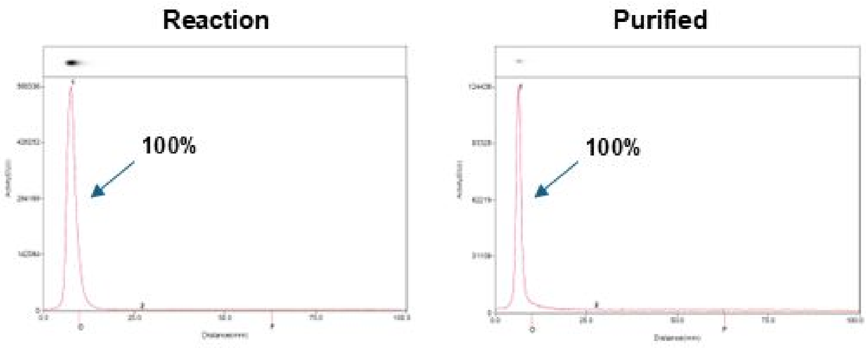

Figure S17. iTLC of the [^{89}Zr]Zr-DFO reaction mixture before and after purification.

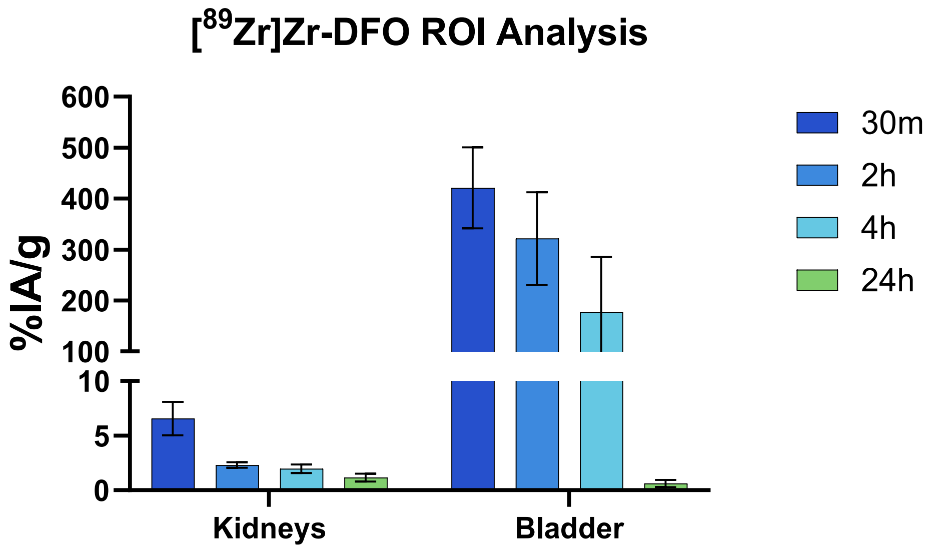

Figure S18. Region of Interest (ROI) quantification of the kidneys and bladder for each mouse at 0.5, 2, 4, and 24h post-injection, reported as the percentage of injected activity per gram of tissue (%IA/g).

Table S11. Region-of-interest (ROI) PET quantification of radiolabeled immune cell biodistribution. Relevant parameters include uptake in lung, liver, spleen, bone, and whole body, reported as percent injected activity per gram of tissue (%IA/g). Measurements were taken at early (1–3 hours) and late (7–8 days) post-injection. Values represent mean ± standard deviation.

Group	*Cell Type*	*N*	*Inj. Activity (µCi)*	*Inj. Cells (×10^5)*	*Lung %IA/g (1–3 h)*	*Liver %IA/g (1–3 h)*	*Bone %IA/g (1–3 h)*	*Whole Body %IA/g (1–3 h)*	*Liver %IA/g (7–8 d)*	*Spleen %IA/g (7–8 d)*	*Bone %IA/g (7–8 d)*	*Whole Body %IA/g (7–8 d)*
Human	U937 Monocytes	3	126–150	3.5–4.1	19.9 ± 0.3	31.5 ± 0.8	0.8 ± 0.02	2.6 ± 0.0	30.0 ± 2.2	12.2 ± 0.6	1.4 ± 0.3	2.5 ± 0.1
Human	PBMCs	2	16–21	1.3–1.7	23.4	31.4	0.8	2.75	34.1	8.4	1.95	2.45
NHP	Neutrophils	3	77–93	12.1–14.6	20.5 ± 2.6	45.0 ± 3.2	0.8 ± 0.4	3.8 ± 0.1	42.4 ± 5.7	11.7 ± 0.5	1.5 ± 0.6	3.3 ± 0.3
NHP	T cells	2	14	8.0	10.9 ± 1.6	26.7 ± 0.8	0.5	3.1	32.4 ± 0.1	7.5 ± 0.1	0.5	2.5
Human	Jurkat T cells	1	69	5.2	17.9	38.2	1.6	4.0	35.2	5.8	1.6	3.4

N = number of mice per group; %IA/g = percent injected activity per gram of tissue; PBMC = peripheral blood mononuclear cell; NHP = non-human primate.

Table S2. Region of Interest (ROI) quantification for [^{89}Zr]Zr-U937 cells in NSG mice (N = 3 except where noted).

[^{89}Zr]Zr-U937s																
**Time (h)**	**Heart (%IA/g)**		**Bone (%IA/g)**		**Kidneys (%IA/g)**		**Liver (%IA/g)**		**Lungs (%IA/g)**		**Muscle (%IA/g)**		**Spleen (%IA/g)**		**Whole Body (%IA/g)**	
	**AVG.**	**S.D.**	**AVG.**	**S.D.**	**AVG.**	**S.D.**	**AVG.**	**S.D.**	**AVG.**	**S.D.**	**AVG.**	**S.D.**	**AVG.**	**S.D.**	**AVG.**	**S.D.**
**3**	1.0	[a]	0.8	[a]	0.9	[a]	23.7	[a]	19.9	[a]	0.1	[a]	6.1	[a]	2.6	[a]
**24**	0.4	0.0	1.1	0.1	0.6	0.1	31.5	0.8	2.4	0.5	0.1	0.0	13.2	3.7	2.6	0.1
**72**	0.3	0.0	1.1	0.2	0.4	0.0	30.2	2.8	1.7	0.2	0.1	0.0	13.5	2.0	2.5	0.1
**168**	0.3	0.1	1.3	0.2	0.3	0.0	28.3	0.6	1.8	0.4	0.1	0.0	13.1	2.0	2.5	0.1

[a]PET data for Mouse 3 at 3h were lost due to data corruption; only data from mice 1 and 2 were quantified at the 3h timepoint.

Table S3. Region of Interest (ROI) quantification for [^{89}Zr]Zr-PBMCs in NSG mice (N = 2).

[^{89}Zr]Zr-PBMCs								
**Time (h)**	**Heart (%IA/g)**	**Bone (%IA/g)**	**Kidneys (%IA/g)**	**Liver (%IA/g)**	**Lungs (%IA/g)**	**Muscle (%IA/g)**	**Spleen (%IA/g)**	**Whole Body (%IA/g)**
	**AVG.**	**AVG.**	**AVG.**	**AVG.**	**AVG.**	**AVG.**	**AVG.**	**AVG.**
**2**	1.23	1.08	1.15	31.35	23.35	0.06	5.90	2.75
**24**	0.51	1.80	0.52	37.45	2.65	0.04	7.75	2.50
**72**	1.25	0.37	0.65	31.85	1.75	0.04	11.25	2.35
**168**	0.20	1.95	0.38	37.50	1.60	0.08	12.40	2.45

Table S4. Region of Interest (ROI) quantification for [^{89}Zr]Zr-NHP-Neutrophils in NSG mice (N = 3).

[^{89}Zr]Zr-NHP-Neutrophils																
**Time (h)**	**Heart (%IA/g)**		**Bone (%IA/g)**		**Kidneys (%IA/g)**		**Liver (%IA/g)**		**Lungs (%IA/g)**		**Muscle (%IA/g)**		**Spleen (%IA/g)**		**Whole Body (%IA/g)**	
	**AVG.**	**S.D.**	**AVG.**	**S.D.**	**AVG.**	**S.D.**	**AVG.**	**S.D.**	**AVG.**	**S.D.**	**AVG.**	**S.D.**	**AVG.**	**S.D.**	**AVG.**	**S.D.**
**3**	4.0	1.1	0.8	0.4	2.5	0.1	45.0	3.2	20.5	2.6	0.3	0.0	8.4	4.8	3.8	0.1
**24**	2.7	1.6	1.4	0.7	2.1	0.8	47.3	6.7	8.4	2.1	0.2	0.1	10.9	3.5	3.6	0.3
**72**	1.9	0.1	1.5	0.5	2.5	0.2	44.6	4.2	7.4	1.0	0.2	0.1	10.3	2.9	3.5	0.2
**168**	1.7	1.3	1.5	0.6	2.5	0.8	42.4	5.7	6.7	3.2	0.2	0.1	11.7	0.5	3.3	0.3

Table S5. Region of Interest (ROI) quantification for [^{89}Zr]Zr-NHP-T cells in NSG mice (N = 2).

[^{89}Zr]Zr-NHP-T cells								
**Time (h)**	**Heart (%IA/g)**	**Bone (%IA/g)**	**Kidneys (%IA/g)**	**Liver (%IA/g)**	**Lungs (%IA/g)**	**Muscle (%IA/g)**	**Spleen (%IA/g)**	**Whole Body (%IA/g)**
	**AVG.**	**AVG.**	**AVG.**	**AVG.**	**AVG.**	**AVG.**	**AVG.**	**AVG.**
**1**	5.0	0.2	1.4	26.7	10.9	0.1	3.6	3.1
**24**	0.5	0.3	1.8	31.6	2.1	0.0	7.6	2.7
**72**	0.3	0.6	1.4	31.5	1.4	0.0	7.3	2.7
**168**	0.3	0.5	1.7	32.4	1.7	0.0	7.5	2.5

Table S6. Region of Interest (ROI) quantification for [^{89}Zr]Zr-Jurkat cells in NSG mice (N = 1).

[^{89}Zr]Zr-Jurkats								
**Time (h)**	**Heart (%IA/g)**	**Bone (%IA/g)**	**Kidneys (%IA/g)**	**Liver (%IA/g)**	**Lungs (%IA/g)**	**Muscle (%IA/g)**	**Spleen (%IA/g)**	**Whole Body (%IA/g)**
	**AVG.**	**AVG.**	**AVG.**	**AVG.**	**AVG.**	**AVG.**	**AVG.**	**AVG.**
**1**	7.3	1.6	2.1	38.2	17.9	0.19	2.2	4
**48**	1.9	1.4	2.4	39.7	7.1	0.17	4.3	3.8
**168**	1.6	0.96	2.1	35.2	4.5	0.27	5.8	3.4

Table S7. Ex vivo quantification for the biodistribution of [^{89}Zr]Zr-U937 cells (N = 3), [^{89}Zr]Zr-PBMCs (N = 2), [^{89}Zr]Zr-NHP-Neutrophils (N = 3), [^{89}Zr]Zr-NHP-T cells (N = 2), and [^{89}Zr]Zr-Jurkat cells (N = 1) in NSG mice.

Tissue Uptake (%IA/g)	[^{89}Zr]Zr-U937s		[^{89}Zr]Zr-PBMCs	[^{89}Zr]Zr-NHP-Neutrophils		[^{89}Zr]Zr-NHP-T cells	[^{89}Zr]Zr-Jurkats
	AVG.	S.D.	AVG.	AVG.	SD.	AVG.	AVG.
Blood	0.0	0.0	0.1	0.1	0.1	0.0	0.0
Skin	0.2	0.1	0.1	0.2	0.0	0.1	0.2
Muscle	0.1	0.0	0.1	0.1	0.0	0.0	0.5
Bone	2.6	0.8	2.6	3.5	0.5	1.2	5.3
Heart	0.1	0.0	0.1	0.3	0.1	0.1	0.2
Lungs	3.6	0.5	4.2	12.7	1.7	3.4	15.7
Liver	40.8	8.9	53.1	65.1	4.6	52.6	56.5
Kidneys	0.6	0.0	0.8	3.8	0.3	2.8	2.7
Spleen	106.0	27.4	83.0	85.5	12.3	32.3	10.2
Pancreas	0.8	0.8	0.1	0.2	0.0	0.5	0.2
Stomach	0.4	0.5	0.2	0.2	0.1	0.1	0.3
Intestine	0.1	0.0	0.1	0.2	0.1	0.1	0.1

**Tail**	0.8	0.6	0.5	2.1	2.1	0.3	0.5
**Brain**	0.0	0.0	0.0	0.0	0.0	0.0	0.0
